# *MYC* paralog-dependent apoptotic priming orchestrates a spectrum of vulnerabilities in small cell lung cancer

**DOI:** 10.1038/s41467-019-11371-x

**Published:** 2019-08-02

**Authors:** Marcel A. Dammert, Johannes Brägelmann, Rachelle R. Olsen, Stefanie Böhm, Niloufar Monhasery, Christopher P. Whitney, Milind D. Chalishazar, Hannah L. Tumbrink, Matthew R. Guthrie, Sebastian Klein, Abbie S. Ireland, Jeremy Ryan, Anna Schmitt, Annika Marx, Luka Ozretić, Roberta Castiglione, Carina Lorenz, Ron D. Jachimowicz, Elmar Wolf, Roman K. Thomas, John T. Poirier, Reinhard Büttner, Triparna Sen, Lauren A. Byers, H. Christian Reinhardt, Anthony Letai, Trudy G. Oliver, Martin L. Sos

**Affiliations:** 1 Molecular Pathology, Institute of Pathology, University Hospital of Cologne, 50937 Cologne, Germany; 2 Department of Translational Genomics, Center of Integrated Oncology Cologne-Bonn, Medical Faculty, University of Cologne, 50931 Cologne, Germany; 3 Center for Molecular Medicine Cologne, University of Cologne, 50931 Cologne, Germany; 4 Else Kröner Forschungskolleg Clonal Evolution in Cancer, University Hospital Cologne, 50931 Cologne, Germany; 5 Department of Oncological Sciences, Huntsman Cancer Institute, University of Utah, Salt Lake City, UT 84112 USA; 6 Institute of Pathology, University Hospital of Cologne, 50937 Cologne, Germany; 7 Dana-Farber Cancer Institute, Harvard Medical School, Boston, MA 02215 USA; 8 Department I of Internal Medicine, University Hospital of Cologne, 50931 Cologne, Germany; 9 Cologne Excellence Cluster on Cellular Stress Response in Aging-Associated Diseases, University of Cologne, 50931 Cologne, Germany; 10 Department of Cellular Pathology, Royal Free Hospital, London, NW3 2QG UK; 11 Theodor Boveri Institute, Biocenter, University of Würzburg, 97074 Würzburg, Germany; 12 Memorial Sloan Kettering Cancer Center, New York, NY 10065 USA; 13 Department of Thoracic and Head & Neck Medical Oncology, University of Texas, MD Anderson Cancer Center, Houston, TX 77030 USA

**Keywords:** Targeted therapies, Small-cell lung cancer, Oncogenes, Genetic engineering

## Abstract

*MYC* paralogs are frequently activated in small cell lung cancer (SCLC) but represent poor drug targets. Thus, a detailed mapping of *MYC*-paralog-specific vulnerabilities may help to develop effective therapies for SCLC patients. Using a unique cellular CRISPR activation model, we uncover that, in contrast to MYCN and MYCL, MYC represses *BCL2* transcription via interaction with MIZ1 and DNMT3a. The resulting lack of *BCL2* expression promotes sensitivity to cell cycle control inhibition and dependency on MCL1. Furthermore, *MYC* activation leads to heightened apoptotic priming, intrinsic genotoxic stress and susceptibility to DNA damage checkpoint inhibitors. Finally, combined AURK and CHK1 inhibition substantially prolongs the survival of mice bearing MYC-driven SCLC beyond that of combination chemotherapy. These analyses uncover *MYC*-paralog-specific regulation of the apoptotic machinery with implications for genotype-based selection of targeted therapeutics in SCLC patients.

## Abstract (web summary)

The expression of oncogenic *MYC* paralogs in small cell lung cancer is mutually exclusive. In this study, the authors show that MYC, but not MYCN or MYCL, represses BCL2, resulting in cells that are uniquely sensitive to apoptosis, and find that CHK1 and AURKA inhibitors may be useful for treating these cancers.

## Introduction

Small cell lung cancer (SCLC) is an aggressive neuroendocrine subtype of lung cancer with a 5-year survival rate of only 6% that lacks effective targeted therapies or predictive markers for patient stratification. Genomic amplification of one of the transcription factor paralogs *MYC*, *MYCN*, or *MYCL* occurs in approximately 20% of SCLC patients^1,2^. *MYC* paralog activation is important for tumorigenesis and tumor maintenance, which would make MYC an ideal target for therapeutic intervention^3–5^. While direct inhibition of MYC has not yet been achieved, *MYC* paralog activation in SCLC induces distinct sensitivity profiles to targeted agents such as Aurora Kinase (AURK) or DNA damage checkpoint inhibitors that are preferentially effective in *MYC*-activated cells^6–9^. At the same time, BH3 mimetics, including drugs directed against the anti-apoptotic factors BCL2 and MCL1, represent an attractive class of inhibitors in SCLC but it remains unclear which molecular factors prime susceptibility to these targets^10^. How overexpression of the individual *MYC* paralogs shapes the spectrum of vulnerabilities in SCLC remains elusive.

We hypothesize that a mechanistic understanding of the phenotypic differences associated with activation of individual *MYC* paralogs may allow the discovery of molecularly defined drug targets in SCLC patients. Using CRISPR/dCas9-mediated *MYC* paralog activation, we uncover a link between MYC signaling and the regulation of the apoptotic machinery with direct implications for the selection of targeted drugs for SCLC patients.

## Results

### MYC activation is associated with low *BCL2* expression

**Fig. 1:**
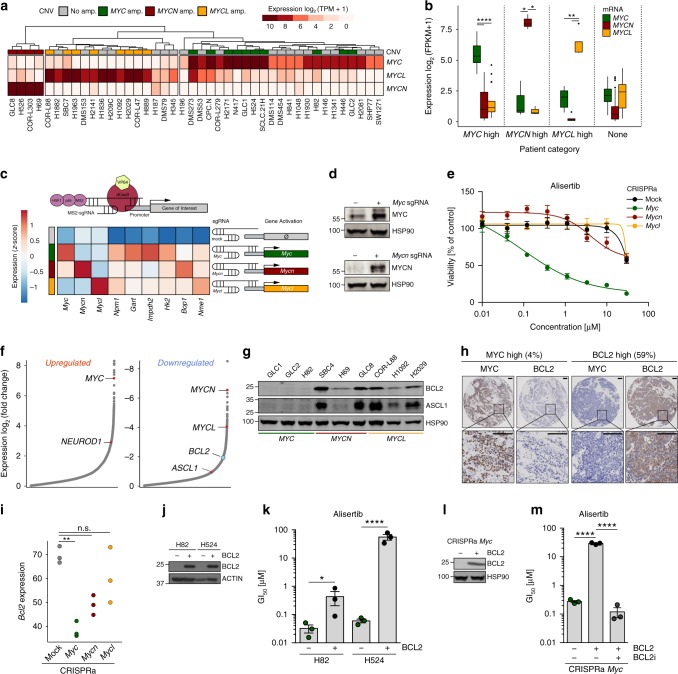
MYC activation is associated with low *BCL2* expression. **a** *MYC* paralog expression (TPM) and copy number variation (CNV) in human small cell lung cancer (SCLC) cell lines (*n* = 42). **b** *MYC* paralog expression in SCLC patients. Center line (median), lower/upper box hinges (25th/75th percentile), whiskers extend to the most extreme value within 1.5× interquartile range (IQR) of the hinges. **c** CRISPRa system for transcriptional upregulation of *Myc* paralogs (top). Expression (*z*-scores) of *Myc* paralogs and Myc target genes in CRISPRa cells (bottom). **d** Western blot showing MYC and MYCN in *Myc-* and *Mycn*-activated CRISPRa cells compared to mock control. HSP90 was used as a loading control. **e** Viability screening of *Myc*-activated CRISPRa cells treated with alisertib for 96 h (*n* = 3). **f** Differentially upregulated and downregulated genes (sorted by log_2_ fold-change) in human SCLC cell lines (*n* = 42) with high *MYC* (*n* = 22) vs. low *MYC* (*n* = 20) expression. **g** Western blot of ASCL1 and BCL2 in *MYC* paralog-amplified human SCLC cell lines (*n* = 9). HSP90 was used as a loading control. **h** Immunohistochemical staining of MYC and BCL2 in tumors of SCLC patients with high *MYC* (left) or high *BCL2* (right) expression (percentage of patients in the cohort (*n* = 49) with high BCL2 or MYC levels is indicated. Scale bar = 100 µm). **i** *Bcl2* expression (counts normalized to library size) in *Myc* paralog-activated CRISPRa cells. Benjamini–Hochberg-adjusted *p* values for *Myc* paralogs were obtained as contrasts of a global differential expression test. **j** Western blot showing BCL2 levels in *MYC*-amplified H82 and H524 cells ± *BCL2* overexpression. HSP90 was used as a loading control. **k** GI_50_ values of *MYC*-amplified H82 and H524 cells ± *BCL2* overexpression treated with alisertib for 72 h (*n* = 3). **l** Western blot of BCL2 in *Myc*-activated CRISPRa cells ± *BCL2* overexpression. HSP90 was used as a loading control. **m** GI_50_ values of *Myc*-activated CRISPRa cells ± *BCL2* overexpression treated with alisertib alone or in combination with 500 nM venetoclax (BCL2i; *n* = 3). Error bars indicate mean ± SEM. Two-tailed unpaired *t* tests, ****p < 0.0001, **p < 0.01, *p < 0.05. Source data are provided as a Source Data file

We analyzed transcriptomes of 42 patient-derived SCLC cell lines and 81 SCLC patient samples^1,6,11^ and found that overexpression of individual *MYC* paralogs is largely mutually exclusive in both datasets (Fig. 1a, b). At the same time, the impact of individual *MYC* paralogs on overall survival remains unclear due to the limited amount of available expression data in SCLC patient cohorts (Supplementary Fig. 1a)^12^. These observations prompted us to dissect the specific role of each *MYC* paralog in SCLC, with the CRISPR/dCas9 Synergistic Activation Mediator (SAM) CRISPR activation (CRISPRa) system^13^ that allows efficient induction of endogenous gene expression. After single guide RNA (sgRNA) selection and validation in NIH3T3 and GEMM-derived (*Trp53/Rb1*-deficient mice, RP) mouse embryonic fibroblasts (MEFs), we activated *Myc*, *Mycn*, or *Mycl* in genomically profiled (whole-exome sequencing (WES)) cells derived from early stage SCLC (RP) tumors^14^ (Supplementary Fig. 1b–d). We observed increased transcription of the individual *Myc* paralogs and elevated MYC and MYCN protein expression (Fig. 1c, d). Although the magnitude of upregulation differed among *Myc* paralogs (Fig. 1c and Supplementary Fig. 1b, c), canonical MYC target genes^6^ were similarly upregulated and proliferation rates were similar between individual cells (Fig. 1c and Supplementary Fig. 1e). However, *Myc-* but not *Mycn-* or *Mycl-*activation induced sensitivity to the AURK inhibitor, alisertib (Fig. 1e), and other cell cycle checkpoint inhibitors (volasertib, *p* = 0.006 mock vs. *Myc*; adavosertib, *p* = 0.05 mock vs. *Myc*, two-tailed unpaired *t* test) similar to patient-derived SCLC cells^6,7^ (Supplementary Fig. 1f).

We next determined differentially expressed genes in *MYC*-high (*n* = 22) vs. *MYC-*low (*n* = 20) human SCLC cell lines (Fig. 1a)^6,7^ to investigate these *MYC*-specific vulnerabilities. Consistent with the *MYC-*associated variant SCLC phenotype, high *MYC* expression correlated with elevated *NEUROD1* (Fig. 1f)^6^. Intriguingly, anti-apoptotic factor *BCL2* was significantly downregulated in *MYC-*overexpressing cells while other BCL2 family members were not differentially expressed (Fig. 1f and Supplementary Fig. 1g, h). In addition, we observed a modest trend toward a negative correlation of *MYC* and *BCL2* in an independent cohort of SCLC patients^15^ (Supplementary Fig. 1i) and significantly decreased *Bcl2* expression in *Myc-*high tumors of *Myc*-driven SCLC mice (RPM) compared to *Trp53/Rb1*-deficient SCLC mouse tumors with low *Myc* expression (Supplementary Fig. 1j)^6^. Furthermore, BCL2 and ASCL1 proteins were only expressed in *MYCN*- and *MYCL*-amplified cells (Fig. 1g and Supplementary Fig. 1k). We observed a similar anti-correlation of MYC and BCL2 protein levels in immunohistochemical (IHC) stainings of human SCLC tumor specimens (*n* = 48) (Fig. 1h and Supplementary Table 1). *Myc* activation also suppressed *Bcl2* expression in CRISPRa cells (*p* = 0.004 mock vs. *Myc*, two-tailed unpaired *t* test) (Fig. 1i). This anti-correlation between MYC and BCL2 appears to be an exception rather than the rule since we primarily found a positive correlation between *MYC* and *BCL2* expression in the pan-cancer CCLE cohort^16,17^ (Supplementary Fig. 1l). Reintroduction of BCL2 strongly reduced sensitivity toward alisertib in both *MYC-*amplified patient-derived cell lines (Fig. 1j, k) and *Myc*-activated CRISPRa cells (Fig. 1l, m). Conversely, co-treatment of *BCL2*-overexpressing *Myc*-activated CRISPRa cells with BCL2-specific inhibitor venetoclax restored the activity of alisertib (Fig. 1m). Of note, exogenous *BCL2* overexpression did not alter cell cycle progression or proliferation rates (Supplementary Fig. 1m, n). Thus *MYC* paralog expression is tightly linked with *BCL2* expression, which determines susceptibility to cell cycle checkpoint inhibitors.

### MYC represses *BCL2* expression

**Fig. 2:**
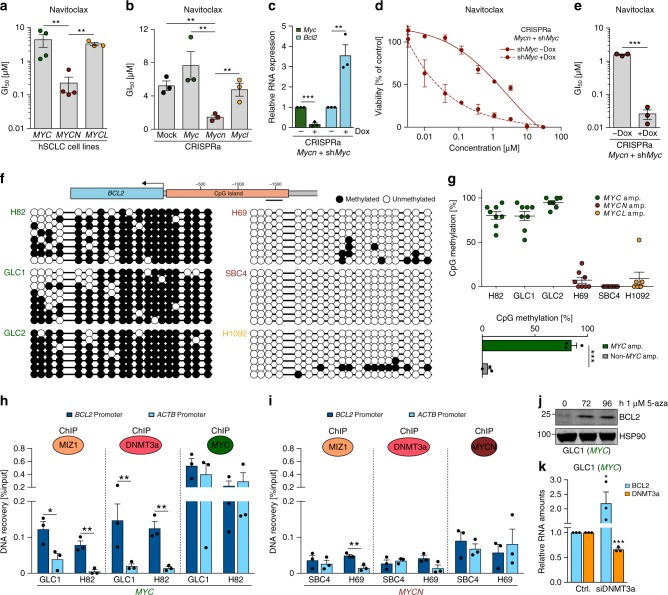
MYC represses *BCL2* expression. **a** GI_50_ values of *MYC* (*n* = 4; GLC1, H82, H524, GLC2), *MYCN* (*n* = 4; GLC8, H69, SBC4, COR-L303), and *MYCL* (*n* = 3; H1092, H2029, SBC7) human small cell lung cancer (SCLC) cell lines treated with navitoclax for 72 h (*n* = 3). **b** GI_50_ values of *Myc* paralog-activated CRISPRa cells treated with navitoclax for 96 h (*n* = 3). **c** Relative mRNA levels (quantitative reverse transcription polymerase chain reaction (qRT-PCR)) of *Myc* and *Bcl2* in *Mycn*-activated CRISPRa cells with Tet-inducible *Myc* knockdown by shRNA at 72 h after doxycycline treatment (*n* = 3). Data were normalized to 18S rRNA. **d** Viability screening of *Mycn*-activated CRISPRa cells after *Myc* knockdown treated with navitoclax for 96 h (*n* = 3). **e** GI_50_ values of viability screening in **d** (*n* = 3). **f** DNA methylation at the *BCL2* promoter (indicated region) measured by bisulfite sequencing. Lollipop diagram representing methylated (black) and unmethylated (white) CpGs in *MYC* (H82: *n* = 8, GLC1: *n* = 8, GLC2: *n* = 7), *MYCN* (H69: *n* = 8, SBC4: *n* = 8), and *MYCL* (H1092: *n* = 7) amplified human SCLC cell lines. **g** Percentage of methylated CpG residues in *MYC* (H82, GLC1, GLC2), *MYCN* (H69, SBC4), and *MYCL* (H1092) amplified human SCLC cell lines (top). CpG methylation percentage (**f**) in SCLC cell lines (*n* = 6) grouped by *MYC* amplification status (*MYC*-high *n* = 3, *MYC*-low *n* = 3; bottom). **h**, **i** Occupancy at the *BCL2* promoter of MYC, MIZ1, and DNMT3a in *MYC*-amplified cells (GLC1, H82) (**h**) and of MYCN, MIZ1, and DNMT3a in *MYCN*-amplified cells (SBC4, H69) (**i**) measured by chromatin immunoprecipitation (ChIP) quantitative real-time PCR (*n* = 3). ChIP signal is displayed as percentage of input. IgG (non-specific antibody control) signal was subtracted from ChIP signal of specific antibodies. **j** Western blot showing BCL2 in *MYC-*amplified GLC1 cells treated with 1 µM 5-azacytidine for the indicated times. HSP90 was used as loading control. **k** Relative mRNA expression (qRT-PCR) of *BCL2* and *DNMT3a* in *MYC*-amplified GLC1 cells treated with control small interfering RNA (siRNA) or *DNMT3a* siRNA (*n* = 3). Data were normalized to 18S rRNA. Error bars indicate mean ± SEM. Two-tailed unpaired *t* tests, ***p < 0.001, **p < 0.01, *p < 0.05. Source data are provided as a Source Data file

As reported previously^10^, *BCL2* expression only partially translated into BCL2 inhibitor activity (Fig. 2a, b and Supplementary Fig. 2a–d). Patient-derived (*n* = 4) and murine CRISPRa cell lines with *MYCN*/*Mycn* overexpression were sensitive to BCL2 inhibitors navitoclax and ABT-737, whereas *MYC*/*Myc*-overexpressing cells were more resistant to BCL2 inhibition (Fig. 2a, b and Supplementary Fig. 2a–d). Since the CRISPRa cells showed an adherent growth phenotype that is associated with basal activation of *Myc* in these cells^6,18^, we performed short hairpin RNA (shRNA)-mediated knockdown of the endogenous *Myc* in *Mycn-*activated CRISPRa cells (Fig. 2c and Supplementary Fig. 2e). *Myc* knockdown induced *Bcl2* expression (Fig. 2c) and increased sensitivity to BCL2 inhibitors (Fig. 2d, e and Supplementary Fig. 2f, g). Since repression of *BCL2* correlates with high DNA methylation at the *BCL2* promoter^19^, we assayed DNA methylation levels of the CpG island within the *BCL2* promoter in human SCLC cell lines. *MYC*-amplified cell lines (*n* = 3) displayed high DNA methylation levels at the *BCL2* promoter (Fig. 2f), whereas *MYCN-* or *MYCL*-amplified cells (*n* = 3) exhibited significantly less DNA methylation in this region indicating active transcription (*MYC*-amplified vs. non-*MYC*-amplified *p* = 0.0001, two-tailed unpaired *t* test; Fig. 2g). Similarly, high *MYC* expression correlated with high levels of *BCL2* promoter methylation in published methylation data of SCLC cell lines (*n* = 65) (Supplementary Fig. 2h)^20^ and patient-derived xenograft SCLC models (Supplementary Fig. 2i, j)^21^. These observations implicate a functional link between high *MYC* expression, increased *BCL2* promoter methylation, and low *BCL2* expression.

MYC was shown to facilitate the establishment of DNA methylation at gene promoters by cooperating with MIZ1 and DNA methyltransferase 3a (DNMT3a)^22^. Using chromatin immunoprecipitation (ChIP) assays, we observed co-occupancy of MYC, MIZ1, and DNMT3a at the *BCL2* promoter (Fig. 2h) with MYC binding at the transcriptionally inactive *BCL2* promoter being as pronounced as at the active *ACTB* promoter. MIZ1 and DNMT3a were enriched only at the *BCL2* promoter in *MYC*-high cells (Fig. 2h). This suggests that MYC/MIZ1/DNMT3a may cooperatively mediate DNA methylation of the *BCL2* promoter (Fig. 2f). In contrast, in *MYCN*-amplified cells only low levels of MIZ1 and DNMT3a were bound to the *BCL2* promoter with no enrichment of DNMT3a compared to the *ACTB* promoter (Fig. 2i). Consistent with previous studies, only MYC but not MYCN or MYCL substantially interacted with MIZ1 (Supplementary Fig. 2k), which is consistent with the model of MYC-specific *BCL2* repression^23,24^. Finally, both pharmacological inhibition of DNA methylation by 5-azacytidine in GLC1 cells as well as small interfering RNA (siRNA)-mediated DNMT3a knockdown in two MYC-amplified SCLC cell lines led to de-repression of *BCL2* (Fig. 2j, k and Supplementary Fig. 2l). Thus DNMT3-mediated DNA methylation may play an important role in the MYC-induced repression of BCL2.

### MYC drives apoptotic priming and MCL1 dependency

**Fig. 3:**
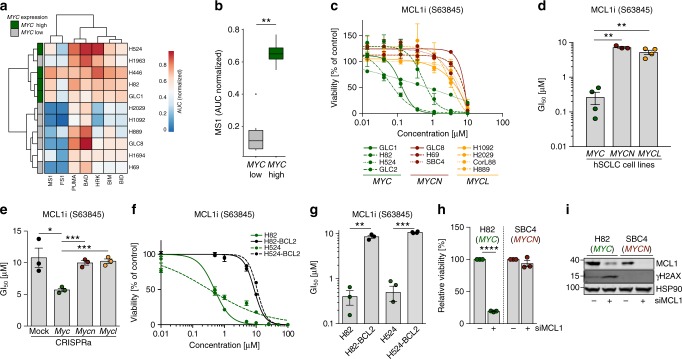
MYC drives apoptotic priming and MCL1 dependency. **a** Heatmap of BH3 profiling showing sensitivity of human small cell lung cancer (SCLC) cell lines against specific apoptosis-inducing peptides. *MYC* expression in the individual cell lines is annotated on the left. **b** Sensitivity of SCLC cell lines toward apoptosis induction by sensitizer peptide MS1 as marker for MCL1-dependent apoptosis. Cell lines are grouped into *MYC* low and high expression. Sensitivity is calculated as area under the curve. Center line (median), lower/upper box hinges (25th/75th percentile), whiskers extend to the most extreme value within 1.5× interquartile range (IQR) of the hinges. **c** Cell viability screening of *MYC* (*n* = 4; GLC1, H82, H524, GLC2), *MYCN* (*n* = 3; GLC8, H69, SBC4), and *MYCL* (*n* = 4; H1092, H2029, CorL88, H889) amplified human SCLC cell lines treated with MCL1 inhibitor (S63845) for 72 h (*n* = 3). **d** GI_50_ values SCLC cell lines treated with S63845. Cell lines are grouped according to their *MYC* status (*n* = 3). **e** GI_50_ values of *Myc* paralog-activated CRISPRa cells treated with S63845. **f** Cell viability screening of *MYC*-amplified H82 and H524 cells ± *BCL2* overexpression treated with S63845 (*n* = 3). **g** GI_50_ values of cell viability screening in **f** (*n* = 3). **h** Relative cell viability of H82 (*MYC*-amplified) and H69 (*MYCN*-amplified) human SCLC cell lines 48 h after transfection with non-targeted small interfering RNA (siRNA) or siRNA directed against MCL1 (*n* = 3). **i** Western blot showing MCL1 and γH2AX levels in H82 (*MYC*-amplified) and H69 (*MYCN*-amplified) human SCLC cell lines 48 h after Ctrl. or MCL1 siRNA transfection. HSP90 was used as a loading control. Error bars indicate mean ± SEM. Two-tailed unpaired *t* tests, ****p < 0.0001, ***p < 0.001, **p < 0.01, *p < 0.05. Source data are provided as a Source Data file

To assess the impact of differential *BCL2* expression on the apoptotic machinery, we performed BH3 profiling^25^ and observed that *MYC*-amplified SCLC cell lines (*n* = 4) were more primed for apoptosis induction (Fig. 3a) especially by MS1 peptide, which acts as an MCL1 antagonist (*MYC*-amplified vs. non-*MYC*-amplified *p* = 0.01, two-tailed unpaired *t* test) (Fig. 3b). Consistently, *MYC*-amplified SCLC cell lines (*n* = 4) were more sensitive to MCL1 inhibitor S63845^26^ compared to *MYCN-* (*n* = 3) and *MYCL-* (*n* = 4) amplified cell lines (*p* = 0.003 *MYC* vs. *MYCN*; *p* = 0.001 *MYC* vs. *MYCL*, two-tailed unpaired *t* test) (Fig. 3c, d and Supplementary Fig. 3a). Despite a lower activity against murine MCL1^27^, we observed an increased susceptibility to S63845 only in *Myc* but not in *Mycn*- or *Mycl*-activated CRISPRa cells (Fig. 3e). We also observed this *MYC*-induced sensitivity against MCL1 inhibition in clonogenic assays (Supplementary Fig. 3b). In line with previous reports, we observed a reduction of MCL1 protein stability upon AURK inhibition (Supplementary Fig. 3c)^28^ potentially contributing to the high alisertib sensitivity of *MYC*-overexpressing cells. *BCL2* overexpression mitigated the effects of MCL1 inhibition (Fig. 3f, g), indicating the importance of *MYC*-induced *BCL2* repression in defining MCL1 dependency and MYC-specific vulnerabilities in SCLC. Consistently, siRNA-mediated MCL1 knockdown reduced viability only in *MYC*-overexpressing cells (Fig. 3h, i) underlining the MYC-induced dependency on MCL1. Interestingly, *MYC-*amplified cells exhibited increased levels of the DNA-damage response (DDR) marker γH2AX upon MCL1 knockdown and MCL1 inhibition (Fig. 3i; Supplementary Fig. 3d).

To determine the effects of BCL2 family inhibition in vivo, we evaluated the efficacy of BCL2 inhibitor venetoclax and MCL1 inhibitor S63845 in an *Myc*-driven SCLC mouse model (RPM)^6^. As expected, BCL2 inhibition had no beneficial effect on overall survival of RPM mice (Supplementary Fig. 3e). While single agent S63845 (25 or 40 mg/kg) and combined S63845/chemotherapy at 25 mg/kg of the MCL1 inhibitor had a modest effect on the survival of RPM mice, the 40 mg/kg S63845 and chemotherapy combination failed to improve survival of the mice beyond vehicle treatment (Supplementary Fig. 3e). While the trend for the higher efficacy of single agent MCL1 vs. BCL2 inhibition is consistent with our in vitro results, the limited affinity of S63845 for murine MCL1^22,27^ may mask otherwise stronger effects in this murine GEMM. In addition, S63845/chemotherapy regimens induced pronounced weight loss indicating high toxicity for the combination that prohibited a dose escalation for the MCL1 inhibitor (Supplementary Fig. 3f). Taken together, lack of BCL2 expression favors a potentially druggable MCL1 dependency in MYC-activated SCLC.

### MYC triggers a druggable DDR in vivo

**Fig. 4:**
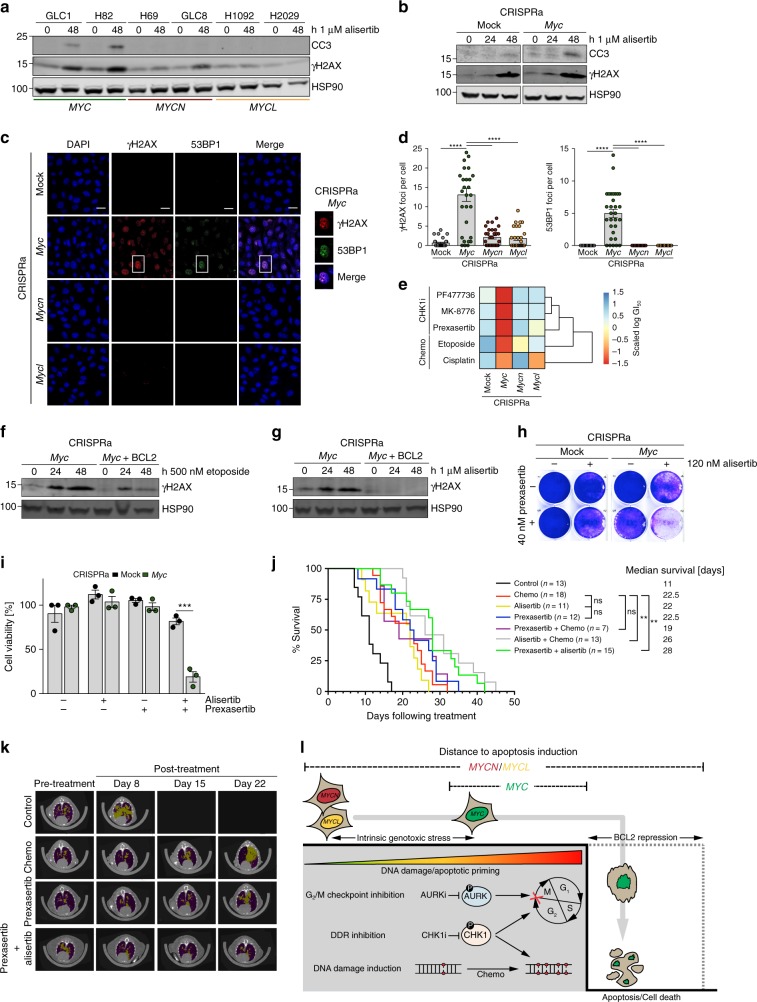
MYC triggers a druggable DNA-damage response (DDR) in vivo. **a**, **b** Western blot of cleaved caspase 3 (CC3) and γH2AX in *MYC*-variant-amplified human small cell lung cancer (SCLC) cell lines (*n* = 6) (**a**) or *Myc*-activated CRISPRa cells (**b**) treated with alisertib for the indicated times. HSP90 was used as a loading control. **c** Representative images of immunofluorescence (IF) experiments of *Myc* paralog-activated CRISPRa cells showing DAPI (DNA), γH2AX (DDR activation), and 53BP1 (DNA double-strand breaks) staining (Scale bar: 20 µm). **d** Quantification of **c** showing mean number of γH2AX (top) and 53BP1 (bottom) foci per cell (*n* = 30). Error bars indicate mean ± SEM. One-way analysis of variance, ****p < 0.0001. **e** Heatmap displaying sensitivity (scaled log(GI_50_)) of *Myc* paralog-activated CRISPRa cells treated with CHK1 inhibitors (MK8776, PF477736, prexasertib) or chemotherapeutics (etoposide, cisplatin) for 96 h (*n* = 3). **f**, **g** Western blot of γH2AX in *Myc*-activated CRISPRa cells ± *BCL2* overexpression treated with etoposide (**g**) and alisertib (**h**). HSP90 was used as a loading control. **h** Crystal violet assay of control and *Myc*-activated CRISPRa cells upon treatment with 120 nM alisertib, 40 nM prexasertib, and combined treatment for 96 h. **i** Viability of mock control and *Myc*-activated CRISPRa cells upon treatment with 120 nM alisertib, 40 nM prexasertib, and combined treatment for 96 h (*n* = 3). Error bars indicate mean ± SEM. Two-tailed unpaired *t* tests, ***p < 0.001. **j** Survival analysis of RPM mice bearing MYC-driven SCLC treated with vehicle control (phosphate-buffered saline (PBS), *n* = 13), chemotherapy (cisplatin/etoposide, *n* = 18), Aurora Kinase (AURK) inhibitor alisertib (n = 11), checkpoint kinase 1 (CHK1) inhibitor prexasertib (*n* = 12), prexasertib+chemotherapy (*n* = 7), alisertib+chemotherapy (*n* = 13), and prexasertib+alisertib (*n* = 15). Log-rank (Mantel–Cox) test, **p < 0.009. **k** Representative micro-computed tomographic images of RPM mice pre-treatment and after treatment with vehicle control (PBS), chemotherapy (cisplatin/etoposide), CHK1 inhibitor prexasertib, and prexasertib combined with AURK inhibitor alisertib. Tumors are colored in yellow, air space in purple. **l** Model of *MYC* paralog-dependent apoptotic priming and vulnerabilities in SCLC. Source data are provided as a Source Data file

Next, we investigated the impact of cell cycle checkpoint inhibition on the induction of DDR and apoptosis. We observed that alisertib as well as volasertib treatment led to a rapid induction of γH2AX and CC3 levels in human *MYC*-amplified and murine CRISPRa *Myc*-activated cells (Fig. 4a, b and Supplementary Fig. 4a, b). Using immunofluorescence, we observed increased steady-state levels of γH2AX (*p* < 0.0001, one-way analysis of variance (ANOVA)) and DNA double-strand break (DSB) marker 53BP1 (*p* < 0.0001, one-way ANOVA) in *Myc-*activated murine SCLC cells suggesting DSB-mediated activation of the DDR in these cells (Fig. 4c, d). Basal DDR activation was also observed in *MYC*-overexpressing human SCLC cells (Supplementary Fig. 4c, d). Further elevation of DNA damage by chemotherapeutics (etoposide and cisplatin) or checkpoint kinase 1 (CHK1) inhibitors (prexasertib, PF-477736, MK-8776)^6,9^ decreased viability preferentially in *Myc-*activated CRISPRa cells (Fig. 4e). Consistently, etoposide treatment of *Myc*-activated cells rapidly induced γH2AX and CC3 levels (Supplementary Fig. 4e). Interestingly, *BCL2* overexpression reduced γH2AX levels after both etoposide and alisertib treatment of *Myc*-activated CRISPRa cells (Fig. 4f, g). We next combined AURK and CHK1 inhibition and observed synergistic activity at low nanomolar concentrations of alisertib and prexasertib in clonogenic and viability assays (Fig. 4h, i) with more pronounced synergy in *Myc*-activated cells (Supplementary Fig. 4f).

In SCLC patients who are routinely treated with first-line cisplatin/etoposide combination chemotherapy, emergence of chemo-resistance is rapid and frequent. To assess the efficacy of combined AURK/CHK1 inhibition in the setting of chemo-resistance, we generated chemo-resistant cell lines from *Myc*-activated CRISPRa cells (Supplementary Fig. 4g) and subjected these cells to combined AURK/CHK1 inhibition. We observed that chemo-resistance substantially increased resistance against combined AURK/CHK1 inhibition (Supplementary Fig. 4h) arguing for efficacy of this treatment strategy in the first-line setting but not upon chemo-resistance. Interestingly, chemo-resistant cells displayed increased AURK and CHK1 phosphorylation levels suggesting higher activity of both enzymes after chemo-resistance (Supplementary Fig. 4i).

We next sought to validate the efficacy of AURK/CHK1 inhibition in vivo in the *Myc*-driven RPM mouse model. Compared to vehicle treatment, CHK1 inhibitor treatment (prexasertib) prolonged survival of RPM mice similar to chemotherapy (cisplatin/etoposide) (Fig. 4j). The combination of prexasertib and AURK inhibitor (alisertib) further prolonged survival of RPM mice compared to chemotherapy (median survival 22.5 vs. 28 days; *p* = 0.005, Log-rank (Mantel–Cox) test; Fig. 4j). Furthermore, the combination of either prexasertib or alisertib with chemotherapy was slightly less effective (median survival 19 and 26 days) than the alisertib/prexasertib combination (Fig. 4j). Vehicle-treated mice exhibited rapid tumor growth while chemotherapy- or prexasertib-treated animals exhibited a modest delay in tumor growth followed by rapid relapse (Fig. 4k and Supplementary Fig. 4j). Of importance, mice treated with the combination of targeted therapy agents, alisertib and prexasertib, exhibited moderate weight loss compared to regimens in which targeted agents were combined with chemotherapy (Supplementary Fig. 4k). This suggests manageable toxicity of combined AURK/CHK1 inhibition and further strengthens our model in which MYC-dependent tumors are more susceptible to perturbation of the cell cycle and DDR control pathways (Fig. 4i).

## Discussion

Here we investigated how *MYC* paralogs modulate drug dependencies in SCLC. We developed an isogenic CRISPRa-based model to study the endogenous activation of the different *MYC* paralogs in GEMM-derived SCLC cell lines. This cellular system allowed us to molecularly define and phenotypically characterize *MYC*-paralog-driven SCLC uncoupled from the divergent genetic background of patient-derived cell lines.

In summary, our data provide mechanistic insight into *MYC*-paralog-specific dependencies with direct implications for a personalized treatment against SCLC tumors. Our findings reveal a pivotal role for BCL2 as a major regulator of response to cell cycle and DNA damage checkpoint inhibitors. In SCLC cells, *MYC* activation represses *BCL2* thereby limiting the pool of anti-apoptotic proteins. Indeed, we observed increased apoptotic priming and a strong MCL1 dependency in *MYC-*overexpressing cells, which are also vulnerable to direct and indirect DNA damage induction (Fig. 4l). Differential MYC protein levels do not alter *MCL1* expression, so lack of BCL2 likely is the main driver of MYC-induced MCL1 dependency. Consistent with previous reports^29^, reintroduction of BCL2 mitigated DNA damage-induced cell death. The specific silencing of an anti-apoptotic protein such as *BCL2* seems to be paradoxical since the benefit to *MYC-*activated cells is not obvious. A study by Ichim and colleagues described limited mitochondrial outer membrane permeabilization (MOMP), a phenomenon termed minority MOMP, as a trigger for cellular transformation and tumorigenesis^30^. In this scenario, limited caspase activity promotes DNA damage induction and genome instability. The steady-state γH2AX levels in cells with high *MYC* strongly resemble such a limited MOMP baseline. Following this hypothesis, suppression of BCL2 may facilitate the induction of this phenotype. On the other hand, increased levels of γH2AX may contribute to DNA damage accumulation in *MYC*-activated cells following chemotherapy and/or CHK1 inhibition. Furthermore, a disruption of the G2/M checkpoint via AURK inhibition, in the background of TP53/RB1-loss-induced defective G1/S checkpoint, may have the same cytotoxic effects. Failure to repair accumulated DNA lesions likely induces apoptosis followed by cell death (Fig. 4l).

We show that this MYC-specific vulnerability can be therapeutically exploited in vitro and in vivo by combined AURK/CHK1 inhibition. A translation of this regimen into a clinical setting might primarily be effective in a first-line setting since chemo-resistant cell lines were also resistant to combined AURK/CHK1 inhibition. The combination of two targeted therapy agents at tolerable doses might overcome the need for pan-toxic chemotherapy. Since the efficacy of combining targeted therapy with chemotherapy was also superior compared to chemotherapy alone, this strategy might prevent or delay the emergence of resistance. We observed less toxicity for combined AURK/CHK1 inhibition, which might overcome previously observed hematological toxicities for alisertib, especially in combination with chemotherapy^31^. Several clinical trials are already evaluating AURK (NCT03216343, NCT03092934, NCT02719691, NCT02134067, NCT01118611) and CHK1 (NCT02735980, NCT02797964, NCT02797977, NCT02873975) inhibitors either as single agents or in combination with chemotherapy in SCLC patients. A recently completed Phase II trial (NCT02038647) that investigated the effects of alisertib in combination with paclitaxel as second-line therapy for SCLC initially reported a significant increase in progression-free survival^32^. Interestingly, retrospective analysis of a subset of patients revealed that the alisertib/paclitaxel combination preferentially improved survival of patients with high MYC protein expression^33^. This is in line with our data that suggest high *MYC* expression is predictive of response to AURK and/or CHK1 inhibition. Therefore, our data may facilitate the selection of patients who particularly benefit from this treatment, reducing unnecessary toxicities. Thus our study bolsters the mechanistic understanding of the role of specific *MYC* paralogs for the fine-tuning of the apoptotic machinery and druggable dependencies in SCLC.

## Methods

### Cell culture

Human SCLC cell lines were obtained from ATCC and verified by STR profiling at the University of Utah DNA sequencing core facility or at the Institute for Forensic Medicine of the University Hospital of Cologne. GLC1, GLC2, H82, H524, GLC8, SBC4, H69, COR-L303, SBC7, COR-L88, MEF, and *Myc*-activated CRISPRa cells were cultured in RPMI; H1092, H2029, and H889 were cultured in HITES; NIH3T3 and HEK293T cells were cultured in DMEM. All media were supplemented with 10% fetal bovine serum, 1% Penicillin/Streptomycin, and 1% L-glutamine. All cells were grown at 37 °C in a humidified atmosphere with 5% CO_2_.

### Reagents

For cell culture studies, drugs were dissolved in dimethyl sulfoxide (DMSO) to a final stock concentration of 10 mM except for prexasertib (4.5 mM). With the exception of prexasertib (MedChemExpress), all compounds were purchased from Selleckchem.

### CRISPR activation

Briefly, sgRNA sequences (see Supplementary Table 2) targeting promoters of *Myc*, *Mycn*, and *Mycl* were obtained from the sgRNA design tool (http://sam.genome-engineering.org/database/, Cas9-Activators with SAM, accessed 12/2015) and cloned into lentiSAMv2. Lentiviral particles of lenti-MS2-p65-HSF1_Hygro and lentiSAMv2 (containing *Myc* paralog sgRNAs) were produced in HEK293T cells co-transfected with pMD.2 and pCMVd.8.9 helper plasmids. Target cells were first transduced with lentiviral particles of lentiMS2-p65-HSF1_Hygro followed by hygromycin selection (400 µg/ml). Selected cells were then transduced with lentiviral particles of lentiSAMv2 followed by blasticidin selection (1.5 µg/ml).

### Cell viability screening

To assess cell viability, cells were plated in 96-well plates in triplicates and compounds were added at 8 decreasing compound concentrations 24 h after seeding. Seventy-two hours later, cell viability was measured via Cell Titer-Glo (CTG) assay (Promega) and was normalized to DMSO-treated controls. Half-maximal growth inhibitory (GI_50_) concentrations of cell viability were inferred by fitting sigmoidal dose–response curves using the Prism 8 software (GraphPad). Data are represented as mean ± SEM and significance was calculated by unpaired Student’s *t* tests.

### Cell proliferation kinetics

In all, 2 × 10^4^ cells were plated in triplicate in one well of a 12-well plate. Cell number was determined daily for 4 consecutive days. Data are presented as mean ± SEM.

### Whole-exome sequencing

DNA from *Myc*-paralog-activated cells was extracted using the Gentra Puregene Tissue Kit (Qiagen) according to the manufacturer’s instructions. Library preparation for exome sequencing was performed with the SureSelectXT Library Prep Kit and the Target Enrichment Kit using the Mouse All Exon Capture ab (Agilent, USA) following the SureSelectXT Automated Target Enrichment Illumina PE Multiplexed Seq protocol. Sequencing was performed with a 2 × 76 bp protocol on a HiSeq4000. Raw sequencing reads were aligned to the mouse reference genome mm10 using BWA-MEM, followed by trimming of overlapping read pairs, and removal of PCR duplicates and secondary alignments. For copy number (CN) analysis, Sclust^34^ is applied to estimate purity-corrected CNs by conditionally optimizing likelihoods of allelic imbalances and read ratios relative to available mouse normal data. All sequencing data will be released upon publication. Sequencing data are deposited at EBI Array Express, accession # E-MTAB-7412.

### Transcriptome data analysis

Human SCLC RNA-seq cell line generated within this study and SCLC cell line raw data used previously^6^ were aligned to the human reference genome Hg38 using STAR^35^ followed by gene expression quantification as transcript per million (TPM) and counts using RSEM^36^. For differential gene expression, cell lines were grouped according to *MYC* expression into *MYC*-high (*n* = 22, COR.L279, CPC.N, DMS114, DMS273, DMS454, DMS53, GLC1, GLC2, H1048, H1341, H1930, H2171, H446, H524, H82, H841, NCI.H146, NCI.H2081, NCI.N417, SCLC.21 H, SHP77, SW1271) and *MYC*-low (*n* = 20, COR.L303,COR.L47, COR.L88, DMS153, DMS79, GLC8, H1836, H196, H1963, H2029, H209C, H2141, H526, H69, H889, NCI.H1092, NCI.H187, NCI.H1882, NCI.H345, SBC7). Differential gene expression between groups was calculated from count-level data using DESeq2^37^. Resulting *p* values were adjusted using Benjamini–Hochberg correction. Annotation of *MYC* paralog amplification status in human SCLC cell lines was obtained from published genomic data^7,20,38^. 3’ RNA-seq data was aligned to the mouse reference genome GRCm38 using STAR and quantified with RSEM prior to downstream analysis. Processed human primary SCLC tumor sample data were acquired from a published study^1^. Primary samples were classified as MYC family member high vs. low based on gene expression, where cut-offs were derived from Gaussian-mixture models. In brief, samples were grouped by fitting two normal distributions to log-transformed expression of the MYC family member. Cut-offs between high and low expression groups were derived using the respective fitted distributions. Publicly available RNAseq data for a cohort of 79 SCLC patients^15^ was obtained from GEO (GSE60052) including normalized log_2_-transformed expression per gene. Patients were categorized in 15 bins based on *MYC* expression. Median expression levels of *MYC* and *BCL2* per bin were calculated and correlated using Spearman correlation coefficient. To assess RNA expression of *Bcl2* in mouse tumor models, we used published expression data including RNAseq of RPM (*n* = 11) and RPR2 (*n* = 4) mouse models^6^, supplemented with gene expression array data for (RP (*n* = 10) and RPP130 (*n* = 3) mouse tumors (GSE18534)^39^. Log_2_-transformed intensity values were averaged per gene if multiple probes were present. To account for potential effects of expression analysis method, log_2_-FPKM values and log_2_-intensity values were transformed to *z*-scores per sample followed by quantile normalization per gene across samples prior to joined analysis. To assess correlation of *MYC* and *BCL2* mRNA expression across various cancer entities, cell line RNAseq data generated by the CCLE was downloaded from www.depmap.org (Release 19Q1). To account for entity-specific baseline expression differences of *MYC* and *BCL2*, log_2_-transformed expression levels quantified as TPM were first scaled per gene within each of the 27 tumor entities before calculating Pearson correlation.

### Cell cycle analysis by flow cytometry

A total of 5 × 10^5^ cells were seeded in 6-well plates and incubated overnight, before addition of 2 mM thymidine for 16 h (first block). After the first block, cells were washed twice with phosphate-buffered saline (PBS) and incubated in growth medium for 8 h before addition of 2 mM thymidine for 16 h (second block). Cells were washed twice with PBS and released. Every 2 h in a period of 12 h, cells were trypsinized, washed with PBS, fixed with 70% ethanol, and incubated for half an hour on ice. Fixed cells were stored at 4 °C for the cell cycle analysis. Ethanol-fixed cells were centrifuged for 5 min at 300 × *g*, washed twice with cold PBS, and centrifuged for 5 min at 300 × *g*. Cells were then incubated with 100 mg/ml DNase-free RNaseA in PBS for 30 min on ice. Next, cells were washed with PBS and incubated with 100 mg/ml propidium iodide (PI) for 30 min at room temperature (RT) in the dark. Finally, cells were analyzed in a flow cytometer (BD Biosciences). PI fluorescence was determined using FL-3 channel, 488 nm. Raw data were analyzed with the FlowJo software.

### MIZ1/MYC co-immunoprecipitation

HEK293T cells were transfected with pcDNA-HA-HA-MYC, pcDNA-HA-MYCN or pcDNA-HA-HA-MYCL in combination with pcDNA-MIZ1. Two days post-transfection, cells were harvested and subjected to MIZ1 IP using anti-MIZ1 antibody (sc-139685, Santa Cruz Biotechnology, 4 µg). Antibody–protein complexes were captured using 20 µl protein G sepharose beads (Santa Cruz Biotechnology). Immunoprecipitates were then analyzed by western blot.

### GDSC methylation data analysis

Publicly available human SCLC cancer cell line data^20^ including gene expression were obtained from http://www.cancerrxgene.org/ (Genomics of Drug Sensitivity in Cancer Project) and corresponding Illumina 450k methylation beta values (GSE68379) were downloaded from www.ncbi.nlm.nih.gov/geo (NCBI Gene Expression Omnibus, both accessed 27 Dec 2017). SCLC cell lines were classified as *MYC* high vs. *MYC* low based on RMA normalized basal *MYC* expression levels as described above. For methylation analyses, CpGs were filtered using a detection *p* value <0.01 followed by removal of probes containing single-nucleotide polymorphisms, non-CpG probes, and cross-reactive probes^40^. Prior to further downstream analysis, beta values were normalized by peak-based correction^41^. Illumina 450k array annotation files were used to select probes in the BCL2 gene body and promoter region.

### Drug combination screening

Cells were plated in a 6 × 6 matrix of wells of a 96-well plate and treated with alisertib and prexasertib in various independent concentration pairs (concentrations were fixed ranging from 40 nM to 3.3 μM for alisertib and from 10 nM to 1.1 µM for prexasertib) for 96 h followed by viability measurement using CTG assay. Results of three replicate experiments were pooled and synergy was calculated applying a Bliss independence model using the R package synergyfinder^42^.

### Cycloheximide shutoff experiments

Cells were seeded and pre-treated with DMSO (control) or 1 µM alisertib for 24 h before addition of 100 µg/ml cycloheximide for 0, 1, 2, 3, and 4 h. Cell lysates were prepared and analyzed by western blot. Protein amounts of MCL1 were calculated by the Image Studio Software (LICOR Biosciences) and normalized to HSP90 amounts.

### shRNA knockdown experiments

shRNA targeting *Myc* (TGTAAGCTTCAGCCATAATTT) was cloned into a Tet-pLKO-puro vector and cotransfected with pMD2.G and pCMVd.8.9 helper plasmids into HEK 293T cells using TransIT-LT1 reagent (Mirus). Forty-eight hours post-transfection, replication-incompetent lentiviruses were collected from the supernatant for infection in the presence of 8 μg/ml polybrene. Twenty-four hours after infection, growth medium was changed and 3 μg/ml puromycin was added for selection. After 5 passages, *Myc* knockdown was induced by addition of doxycycline (500 ng/ml) and *Myc* knockdown confirmed by RT-qPCR and immunoblot. For compound screenings, doxycycline was added when cells were plated.

### siRNA knockdown experiments

siRNA pools targeting *MCL1* (siMCL1#1 GGUUUGGCAUAUCUAAUAA, siMCL1#2 GAAGGUGGCAUCAGGAAUG, siMCL1#3 GAUUAUCUCUCGGUACCUU, siMCL1#4 CGAAGGAAGUAUCGAAUUU), or *DNMT3a* (siDNMT3A#1 GCAUUCAGGUGGACCGCUA, siDNMT3A#2 GCACUGAAAUGGAAAGGGU, siDNMT3A#3 CUCAGGCGCCUCAGAGCUA, siDNMT3A#4 GGGACUUGGAGAAGCGGAGS) were purchased from Dharmacon and transfected at 20 nM final concentration into SCLC cell lines (H82, SBC4, GLC1, GLC2) using Dharmafect Transfection Reagent #2 (Dharmacon). Growth medium was changed after 12 h. Experiments assessing knockdown efficiency, cell viability, gene expression, and immunoblots to determine knockdown effects were performed 48 h post-transfection.

### Protein overexpression experiments

Vectors pMSCV-PIG (puro-IRES-GFP) and pMSCV-PIG-BCL2 were cotransfected with pMD.2 and pCMVd.8.9 helper plasmids into HEK 293T cells using TransIT-LT1 reagent (Mirus), respectively. Forty-eight hours post-transfection, replication-incompetent lentiviruses were collected from the supernatant for infection of *Myc* CRISPRa cells and H82 and H524 cells in the presence of 8 μg/ml polybrene. Twenty-four hours after infection, growth medium was changed and 3 μg/ml (*Myc* CRISPRa cells) or 1 µg/ml (H82/H524) puromycin was added for selection for the duration of 6 days (3 passages). After selection, cells were analyzed for protein expression.

### RNA isolation qRT-PCR

Total RNA was isolated using the Qiazol reagent (Qiagen) according to the manufacturer’s instructions. In all, 1.5 µg of total RNA was subjected to DNaseI (Sigma) digestion and reverse transcribed using SuperscriptIII (Thermo Fisher Scientific) with random hexamer primers. Quantitative real-time PCR (qPCR) was performed using 7900HT Real-Time PCR System (Applied Biosystems) and the Power SYBR Green PCR Master Mix (Thermo Fisher Scientific). The qPCR primers used to analyze mRNA levels are listed in Supplementary Table 2. Data were normalized to 18S rRNA levels and are presented as mean ± SEM and significance was calculated by unpaired Student’s *t* tests.

### RNA sequencing

Total RNA was isolated using the RNeasy Mini Prep Kit (Qiagen) according to the manufacturer’s instructions with a 75-bp paired-end protocol on a HiSeq4000 (Illumina, USA). 3’ UTR RNA sequencing libraries for murine CRISPRa cells were prepared using the QuantSeq 3’ mRNA-Seq Library Kit (Lexogen, Austria) and sequenced with a 50-bp single-end protocol on an Illumina HiSeq4000 (Illumina, USA). Sequencing data are deposited at EBI Array Express, accession # E-MTAB-7411.

### Chromatin immunoprecipitation

Cells were crosslinked in 1% formaldehyde, and chromatin was extracted and sonicated. Equal amounts of chromatin were incubated overnight with specific antibodies against MYC (clone 9E11, ab56, Abcam, 5 µg), MYCN (clone B8.4.B, sc-53993, Santa Cruz Biotechnology, 4 µg), DNMT3a (ab2850, Abcam, 4 µg), MIZ1 (clone 10E2, Elmar Wolf, Würzburg, 15 µl anti-serum), or unspecific mouse IgG (sc-2025, Santa Cruz Biotechnology, 4 µg). ChIP complexes were captured using protein G Dynabeads (Thermo Fisher Scientific), washed, eluted, and decrosslinked. DNA was purified using the ChIP DNA Clean & Concentrator Kit (Zymo Research) and analyzed by qRT-PCR using primers listed in Supplementary Table 2. ChIP signals of non-specific background (IgG) were subtracted from specific antibody ChIP signals. ChIP signals were calculated as percentage of input. Data are presented as mean ± SEM and significance was calculated by unpaired Student’s *t* tests.

### BH3 profiling assay

Cells were pelleted, washed in PBS, resuspended in MEB2 buffer, and 1 × 10^4^ to 2 × 10^4^ cells were added to each well of a 384 non-binding plate containing MEB2 + 20 µg/ml digitonin + sensitizer peptides at 2× the final concentration. Permeabilized cells were incubated for 1 h at RT in the presence of peptides, fixed by the addition of formaldehyde to 1% final concentration for 10 min at RT, and neutralized by the addition of N2 buffer (Tris/glycine) to terminate fixation. Cells were stained overnight by adding Alexa647–Cytochrome C (clone 6H2.B4, Biolegend) to 250 ng/ml final concentration and Hoechst 33342 to 1 µg/ml final concentration. Analysis was conducted on a BD Fortessa or BD Fortessa X20 with gating on DAPI+ singlets and normalization of the Cytochrome C mean fluorescent intensity values to the buffer alone and 25 µM alamethicin controls.

### Immunofluorescence

Murine *Myc* paralog-activated cells were grown on glass coverslips and human SCLC cells were grown on NuncTM Lab-TekTM coated with Gelatine solution 0.1% in PBS (PAN Biotech). Cells were fixed with 4% paraformaldehyde at RT, permeabilized in PBS containing 0.25% Triton X-100, and blocked in PBS containing 0.2% Tween 20 and 3% bovine serum albumin. Cells were incubated overnight with primary antibodies to γH2AX (#05-636, Merck, 1:500), MCL-1 (sc-819, Santa Cruz Biotechnology, 1:100), or 53BP1 (MAB3802, Merck, 1:500). After washing, cells were incubated with secondary antibodies conjugated to Alexa Fluor-488 (A11029, Thermo Fisher Scientific, 1:1000) and Alexa Fluor-647 (A32733, Thermo Fisher Scientific, 1:1000) in combination with DAPI (4′,6-diamidino-2-phenylindole; Sigma, 1:1000). Coverslips were mounted using Fluromount-GTM (Thermo Fisher Scientific). Microscopy was performed using a Zeiss Meta 710 confocal microscope and images were analyzed by the ImageJ software.

### Bisulfite sequencing

Cellular DNA was extracted using the Puregene Kit (Qiagen) according to the manufacturer’s instructions. Five hundred nanograms of DNA were bisulfite converted using the EZ DNA Methylation-Gold Kit (Zymo Research) according to the manufacturer’s instructions. Bisulfite-converted DNA was subjected to methylation-specific PCR using specific primers for the *BCL2* promoter listed in Supplementary Table 2. PCR product was resolved on a 2% agarose gel and purified using the Monarch DNA Gel Extraction Kit (New England BioLabs), cloned into pCR4-TOPO TA Vector (Thermo Fisher Scientific), transformed into XL10-Gold Ultracompetent Cells (Agilent Technologies), and plated onto ampicillin selection LB-agar plates. DNA of single colonies was extracted using the NucleoSpin Plasmid EasyPure Mini Kit (Macherey-Nagel) and submitted to Sanger sequencing using sequencing primers M13-for and M13-rev (see Supplementary Table 2). Obtained sequences were analyzed and DNA methylation plots were generated using the QUMA quantification tool for methylation analysis^43^.

### Crystal violet assay

In all, 2 × 10^5^ cells were plated into one well of a 6-well plate and treated with DMSO (control), 40 nM prexasertib, 120 nM alisertib, and the combination of prexasertib and alisertib. Seventy-four hours after treatment, cells were fixed in 4% paraformaldehyde in PBS, stained with 0.1% crystal violet in PBS, and rinsed in PBS before image acquisition.

### Generation of chemo-resistant cells

*Myc*-activated CRISPRa cells were subjected to prolonged etoposide treatment at increasing concentrations starting from 500 nM for several weeks. The resulting, proliferating cell line was maintained in growth medium containing 2 µM etoposide.

### Mouse drug treatments

To initiate lung tumors Rb1^fl/fl^;p53^fl/fl^;Myc^LSL/LSL^ (RPM) mice were infected by intratracheal injection with 1 × 10^8^ Ad-CGRP-Cre virus (University of Iowa Virus Vector Core). Mice were imaged with a Quantum FX or GX2 microCT system (Perkin Elmer) and randomized into treatment groups upon detection of ~10% lung tumor burden. Treatment groups included PBS control (*n* = 15), chemotherapy (cisplatin/etoposide, *n* = 18), prexasertib (*n* = 12), or prexasertib combined with alisertib (*n* = 15). Prexasertib (10 mg/kg in Captisol) was administered via subcutaneous flank injection twice a day on a weekly schedule of 2 days on and 5 days off. Prexasertib was provided by Dr. Lauren Byers and manufactured by the Institute for Applied Chemical Science at MD Anderson, Houston, TX. Alisertib (Apexbio Technology; 20 mg/kg in 10% β-cyclodextrin) was administered via oral gavage twice a day on a weekly schedule of 5 days on and 2 days off. For weekly chemotherapy treatments, cisplatin (Sigma-Aldrich; 5 mg/kg in PBS) was administered on day 1 and etoposide (Sigma-Aldrich; 10 mg/kg in 70% PEG in water) was given on day 2 by intraperitoneal injection. To decrease toxicity, mice treated with prexasertib and chemotherapy received cisplatin on day 1, etoposide on day 2, and prexasertib on days 5 and 6 of each weekly cycle. After 4 cycles of cisplatin/etoposide chemotherapy, mice were treated weekly with etoposide only. MCL1 inhibitor S63845 (25 mg/kg or 40 mg/kg in 20% β-cyclodextrin with 25 mM HCl) was administered by tail vein injection. Mice were treated with MCL1i at 25 mg/kg twice/week or 40 mg/kg once/week, and both treatment doses were tested in combination with cisplatin/etoposide chemotherapy. Since neither the 25 mg/kg nor 40 mg/kg monotherapy significantly improved survival, these groups were combined for data analysis. Both tested doses of S63845 + chemotherapy induced significant weight loss and toxicity. ABT-199 (50 mg/kg in 60% Phosal50, 30% PEG400, 10% ethanol) was administered by oral gavage once per day on a weekly schedule of 5 days on/2 days off. Mice were imaged at the start of each treatment cycle and 4 days post cisplatin, and images were quantified using the Analyze 11.0 (AnalyzeDirect) software. Endpoints for survival studies included labored breathing, >20% weight loss, or signs of toxicity. Mice were sacrificed via CO_2_ asphyxiation prior to necropsy. Survival curve analysis was performed with the GraphPad Prism software. These experiments were approved by the HCI Institutional Animal Care and Use Committee (IACUC), and mice were housed in a specific pathogen-free barrier facility.

### Micro-computed tomographic (microCT) imaging

Mice were scanned for 34 s under isoflurane anesthesia using a small animal Quantum FX or GX2 microCT (PerkinElmer) at 45 µm resolution, 90 kV, with 160 mA current. Images were acquired using the PerkinElmer Quantum FX software and processed with Analyze 11.0 (AnalyzeDirect). Scans were calibrated for Hounsfield Units (HU) by determining the mean value of “Bed” and “Air” for representative scans using the region of interest (ROI) tool. Those values were matched to their known HU (40 and −1000 HU, respectively) by the “Image Algebra” tool. A 3 × 3 × 3 Median Filter was applied to every image using the “Spatial Filters” window. Thresholds for “Air” vs. “Dense Tissue” were set using the ROI and histogram tools. For total tumor burden analyses, an object map was created using the previously established thresholds and manually adjusted using “Spline Edit”, “Draw”, “Trace”, and “Nudge Edit” tools. The object map was then morphed, i.e., made binary by using the threshold morphing tool. Then the map was dilated 3 times using 5 × 5 × 5 Jack-shaped structuring elements. Holes were filled on every two-dimensional orientation and the map was finally brought back to its original size with the “Erode” tool 3 times using 5 × 5 × 5 Jack-shaped structuring elements. The volumetric analyses were then performed in the ROI window using the pre-established thresholds and non-airspace was calculated using the formula: Nonairspace = 1 − (VolAir/ROIVol).

### Immunohistochemistry

Tissues were fixed in formalin overnight, then transferred to 70% ethanol, and embedded in paraffin (ARUP histology core). Formalin-fixed paraffin-embedded sections (4 micron) were used for hematoxylin and eosin and IHC staining. Antigen retrieval was performed by boiling slides for 20 min in 0.01 M citrate buffer, pH 6.0. Slides were blocked for 15 min with 3% H_2_O_2_, followed by 5% goat serum in PBS containing 0.1% Tween-20 (PBST). Primary antibodies were incubated overnight at 4 °C and include the following: BCL2 (#M088701-2, clone 124, Agilent), MYC (ab32072, Abcam), NEUROD1 (ab205300, Abcam), and ASCL1 (#556604, BD Pharmingen). Slides were then incubated with horseradish peroxidase (HRP)-conjugated secondary antibody (Vector Laboratories, 1:200) and developed with DAB (Vector Laboratories). A Nikon Eclipse Ci microscope and DS-Fi3 camera were used for imaging.

### Immunoblot

Cell lysates were prepared using RIPA buffer supplemented with protease inhibitors (cOmplete Mini Protease Inhibitor Cocktail, Roche). Protein concentration was determined by BCA assay (Pierce) and equal amounts of protein were separated on 4–20% Tris-glycine sodium dodecyl sulfate-polyacrylamide gel electrophoresis (SDS-PAGE) gels (Thermo Fisher Scientific) and transferred to PVDF-FL membrane (Millipore). Membranes were blocked in 3% cold-fish gelatin blocking buffer in TBS, incubated with primary antibodies, washed, and incubated with fluorescently labeled secondary antibodies prior to detection with Odyssey CLx imaging system (LI-COR Biosciences). Images were processed using the Image Studio Software (LI-COR Biosciences). Primary antibodies are: MYC (#9402, Cell Signaling Technology, 1:1000), MYCN (sc-53993, Santa Cruz Biotechnology, 1:1000), MYCL (AF4050, R&D Systems, 1:1000), BCL2 (#2872, Cell Signaling Technology, 1:1000), BIM (#2933, Cell Signaling Technology, 1:1000), BAD (#610391, BD Biosciences, 1:1000), BCL-XL (#2764, Cell Signaling Technology, 1:1000), HA (#3724, Cell Signaling Technology, 1:1000), MIZ1 (clone 10E2, Elmar Wolf, Würzburg, 1:500), ASCL1 (#556604, BD Biosciences, 1:1000), MCL1 (sc-819, Santa Cruz Biotechnology, 1:1000), γH2AX (#05-636, Merck, 1:1000), Cleaved Caspase 3 – CC3 (#9664, Cell Signaling Technology, 1:500), pAURKA/B/C (#2914, Cell Signaling Technology, 1:1000), pCHK1^S345^ (#2341, Cell Signaling Technology, 1:1000), and HSP90 (ADI-SPA-835, Enzo Life Sciences, 1:5000). Secondary antibodies are: goat anti-rabbit 800CW (#926-32211, LI-COR Biosciences, 1:10,000), goat anti-mouse 800CW (#926-3220, LI-COR Biosciences, 1:10,000), anti-rat 680 (#925-68029, LI-COR Biosciences, 1:10,000), goat anti-rabbit 680LT (#926-68021, LI-COR Biosciences, 1:10,000), and goat anti-mouse 680LT (#926-68020, LI-COR Biosciences, 1:10,000). Alternatively, cells were lysed in RIPA buffer supplemented with Pierce Protease inhibitors and sodium orthovanadate. Protein concentrations were measured with the DC protein assay (Bio-Rad), and equal protein volumes were resolved on SDS-PAGE gels. Samples were transferred to 0.2 µm PVDF (Bio-Rad). Membranes were blocked in 5% milk/PBS-T prior to overnight incubation in primary antibody. Membranes were then incubated in secondary anti-rabbit-HRP or anti-mouse-HRP antibody (Jackson ImmunoResearch, 1:4000). After washing, membranes were developed with WesternBright ECL HRP (Advansta) and imaged on Hyblot autoradiography film. Primary antibodies used include the following: BCL2 (#2872, Cell Signaling Technology, 1:2000); MCL1 (#94296, Cell Signaling Technology, 1:2000); HSP90 (#4877, Cell Signaling Technology, 1:2000); and ACTIN (#A2066, Sigma, 1:10,000). Uncropped blots are displayed in the Source Data file.

### Reporting summary

Further information on research design is available in the Nature Research Reporting Summary linked to this article.

## Supplementary information

Supplementary Information

Reporting Summary

Source Data

## Data Availability

All data supporting the findings in this study are available from the corresponding author upon reasonable request. The primary data underlying the graphs are provided in the Source Data File. Previously published datasets used in this study are available at Gene Expression Omnibus through accession codes GSE60052 (expression data SCLC patients) and GSE68379 (methylation data) and at European Genome-phenome Archive through accession codes EGAS00001002115 and EGAS00001000334 (both RNAseq human SCLC cell lines). RNAseq and WES data generated in this study have been deposited at EBI Array Express with the accession codes E-MTAB-7410 (RNAseq NCI-H69, COR-L303), E-MTAB-7411 (RNAseq rp181 CRISPRa), and E-MTAB-7412 (WES rp181).
