# Supplementary material for: MYC paralog-dependent apoptotic priming orchestrates a spectrum of vulnerabilities in small cell lung cancer
Source: Nat Commun. 2019 Aug 2;10:3485. doi: 10.1038/s41467-019-11371-x (PMC6677768; doi:10.1038/s41467-019-11371-x)
Supplement: Supplementary file 1 — Supplementary Information [file 41467_2019_11371_MOESM1_ESM.pdf]

Supplementary Information

MYC paralog-dependent apoptotic priming orchestrates a spectrum of vulnerabilities in small cell lung cancer

Dammert et al.

Supplementary Figure 1

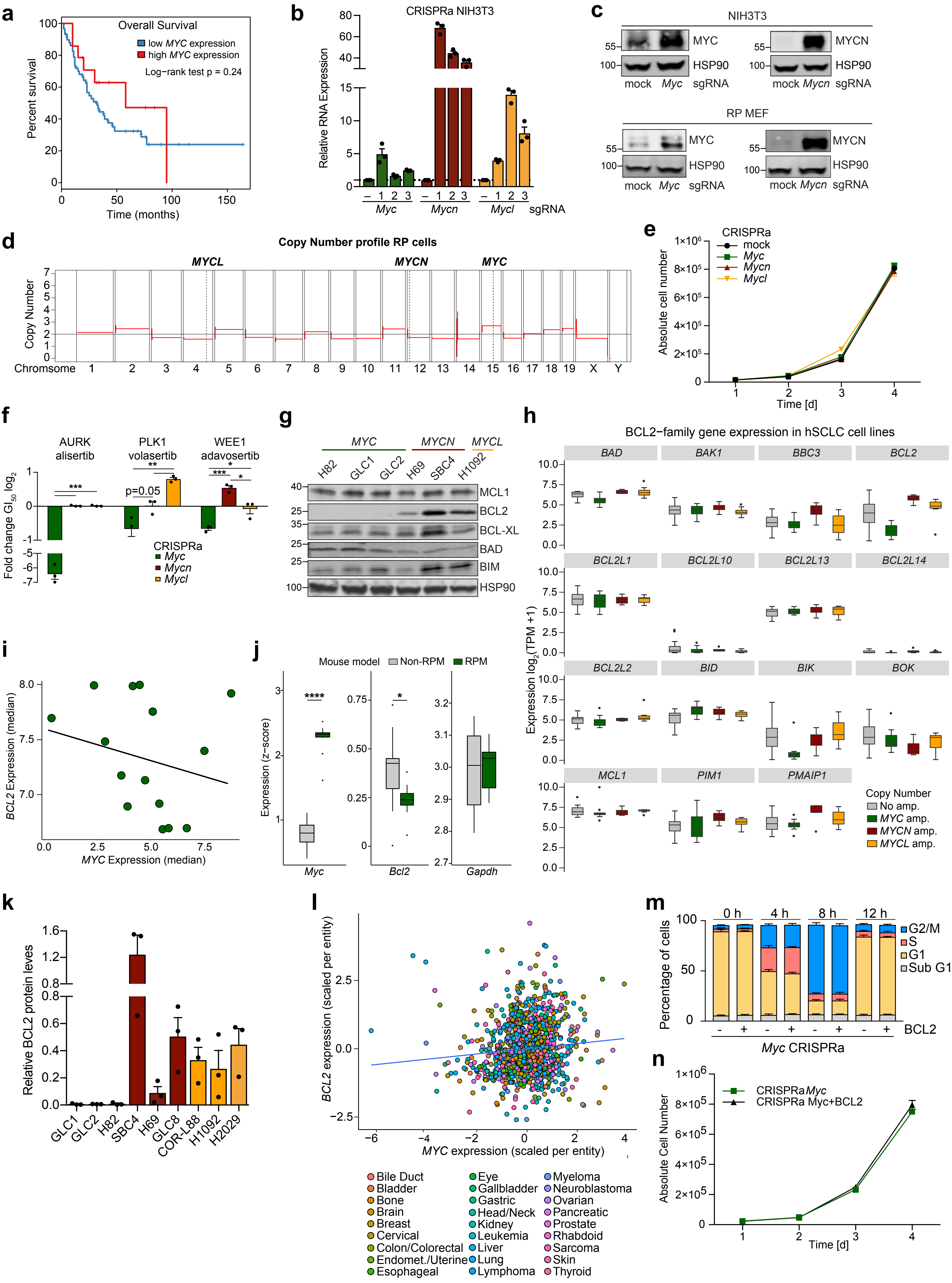

Supplementary Figure 1: MYC activation is associated with low *BCL2* expression

(**a**): Overall survival of SCLC patients (n = 77) grouped according to *MYC* expression.

(**b**): Relative mRNA levels of *Myc* paralogs in *Myc* paralog-activated NIH3T3 cells (n = 3). Three different sgRNAs per *Myc* paralog were tested. RNA expression was normalized to 18S rRNA; Mean ± SEM.

(**c**): Western blot of MYC and MYCN protein levels in *Myc*- and *Mycn*-activated NIH3T3 and RP MEF cells compared to control cells. HSP90 was used as loading control.

(**d**): Copy number profile determined by whole exome sequencing (WES) of RP cells derived from *Trp53/Rb1*-deficient GEM models of SCLC.

(**e**): Cell proliferation of *Myc* paralog-activated CRISPRa cells displayed by absolute cell number at the indicated time points (n = 3). Error bars indicate mean ± SEM.

(**f**): Fold change of GI_{50} values (Log_2 fold change) of *Myc* paralog-activated CRISPRa cells compared to mock control cells treated with AURK inhibitor alisertib, PLK1 inhibitor volasertib and WEE1 inhibitor adavosertib for 96 h (n = 3). Error bars indicate mean ± SEM. Two-tailed unpaired t tests, ***$p < 0.001$, **$p < 0.01$, *$p < 0.05$.

(**g**): Western blot of pro- and anti-apoptotic proteins (MCL1, BCL2, BCL-xL, BAD, BIM) in human SCLC cell lines with different *MYC* paralog amplifications. HSP90 was used as loading control.

(**h**): *BCL2*−family gene expression in human SCLC cell lines determined by RNA-seq. center line = median, lower/upper box hinges = 25th/75th percentile, whiskers extend to the most extreme value within 1.5x interquartile range (IQR) of the hinges.

(i): Correlation of *MYC* and *BCL2* expression (median expression) across a cohort of 79 SCLC patients. r = -0.24.

(j): *Myc*, *Bcl2*, and *Gapdh* expression (z-scores) in *Myc*-driven (RPM) and non-*Myc*-driven (non-RPM) SCLC mouse models. Two-tailed unpaired t tests, Bonferroni-Holm adjusted, ****$p < 0.0001$, *$p < 0.05$. center line = median, lower/upper box hinges = 25th/75th percentile, whiskers extend to the most extreme value within 1.5x interquartile range (IQR) of the hinges.

(**k**): Quantification (n = 3) of BCL2 protein levels in human SCLC cell lines (n = 9, *MYC*-amplified: GLC1, GLC2, H82; *MYCN*-amplified: SBC4, H69, GLC8; *MYCL*-amplified: COR-L88, H1092, H2029) determined by Western blot (**Fig. 1g**). BCL2 levels were normalized to HSP90 (loading control).

(**l**): Correlation of *MYC* and *BCL2* expression (scaled expression per entity) across 1100 CCLE cell lines of the indicated cancer entities.

(**m**): Cell cycle progression of *Myc*-activated CRISPRa cells ± BCL2 overexpression after thymidine block (n = 3).

(**n**): Cell proliferation of *Myc*-activated CRISPRa cells ± BCL2 overexpression displayed by absolute cell number at the indicated time points (n = 3). Error bars indicate mean ± SEM.

Source data are provided as a Source Data file.

Supplementary Figure 2

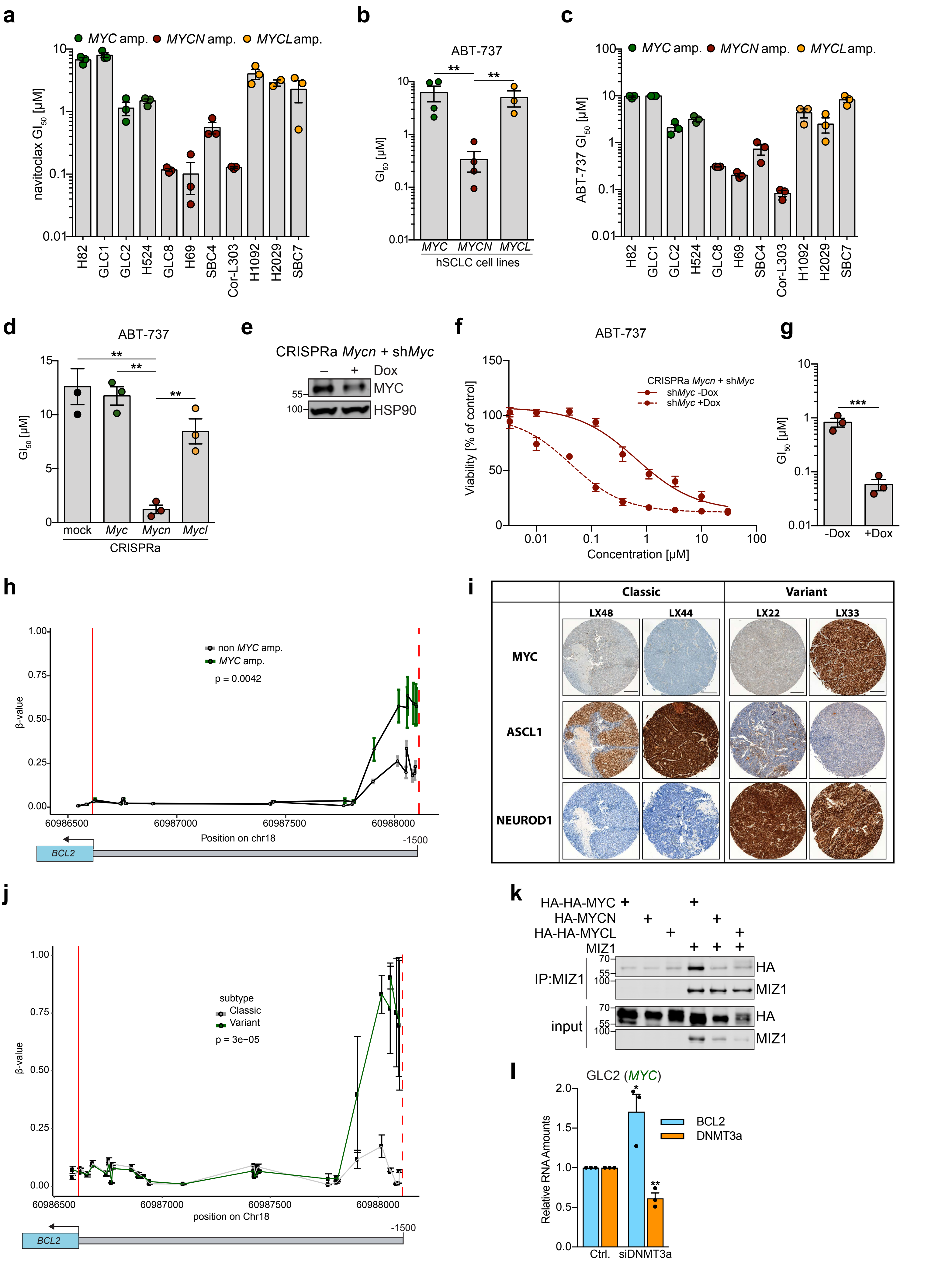

Supplementary Figure 2: MYC represses *BCL2* expression

(**a**): GI_{50} values of individual *MYC*- (n = 4; GLC1, H82, H524, GLC2), *MYCN*- (n = 4; GLC8, H69, SBC4, COR-L303), and *MYCL*- (n = 3; H1092, H2029, SBC7) human SCLC cell lines treated with navitoclax for 72 h (n = 3).

(**b/c**): GI_{50} values of *MYC*- (n = 4; GLC1, H82, H524, GLC2), *MYCN*- (n = 4; GLC8, H69, SBC4, COR-L303), and *MYCL*- (n = 3; H1092, H2029, SBC7) human SCLC cell lines treated with ABT-737 for 72 h (n = 3).

(**d**): GI_{50} values of *Myc* paralog-activated CRISPRa cells treated with ABT-737 for 96 h (n = 3). Error bars indicate mean ± SEM. Two-tailed unpaired t tests, **p < 0.01.

(**e**): Western blot showing protein levels of MYC in *Mycn*-activated CRISPRa cells with Tet-inducible *Myc* shRNA knockdown. shRNA expression was induced by doxocycline for 72 h. HSP90 was used as loading control.

(**f**): Cell viability screening of *Mycn*-activated CRISPRa cells with Tet-inducible *Myc* shRNA knockdown treated with ABT-737 for 96 h (n = 3).

(**g**): GI_{50} values of cell viability screening in (f; n = 3). Error bars indicate mean ± SEM. Two-tailed unpaired t tests, ***p < 0.001.

(**h**): DNA methylation levels of human SCLC cell lines grouped by *MYC* amplification status at *BCL2* transcription start site (TSS, red line) and the *BCL2* promoter region (-1500 bp upstream, dashed red line) represented by β-values from Illumina 450k methylation arrays (Iorio et al., 2016).

(**i**): IHC staining of MYC, ASCL1 and NEUROD1 in SCLC PDX samples of classic (LX48, LX44) and variant (LX22, LX33) SCLC subtypes. Scale bar: 100 µm.

(**j**): DNA methylation levels of SCLC PDX samples of classic (LX48, LX44) and variant (LX22, LX33) SCLC subtypes at *BCL2* transcription start site (TSS, red line) and the *BCL2* promoter region (-1500 bp upstream, dashed red line) represented by β-values from 450 k methylation arrays.

(**k**): Western blot of MIZ1 Co-IP experiments. MIZ1 and HA-tagged versions of the individual MYC family members (MYC, MYCN, MYCL) were transiently overexpressed in HEK293T cells followed by MIZ1 IP. Western blot shows levels of MIZ1 (MIZ1 antibody) and the individual MYC family members (HA antibody) in input and MIZ1 immune precipitate.

(**l**): Relative mRNA expression of *BCL2* and *DNMT3a* in *MYC*-amplified GLC2 cells subjected to non-target control siRNA or siRNA directed against *DNMT3a* (n = 3). RNA amounts were determined by qRT-PCR and normalized to 18S rRNA.

Error bars indicate mean ± SEM. Two-tailed unpaired t tests, ***p < 0.001, **p < 0.01, *p < 0.05. Source data are provided as a Source Data file.

Supplementary Figure 3

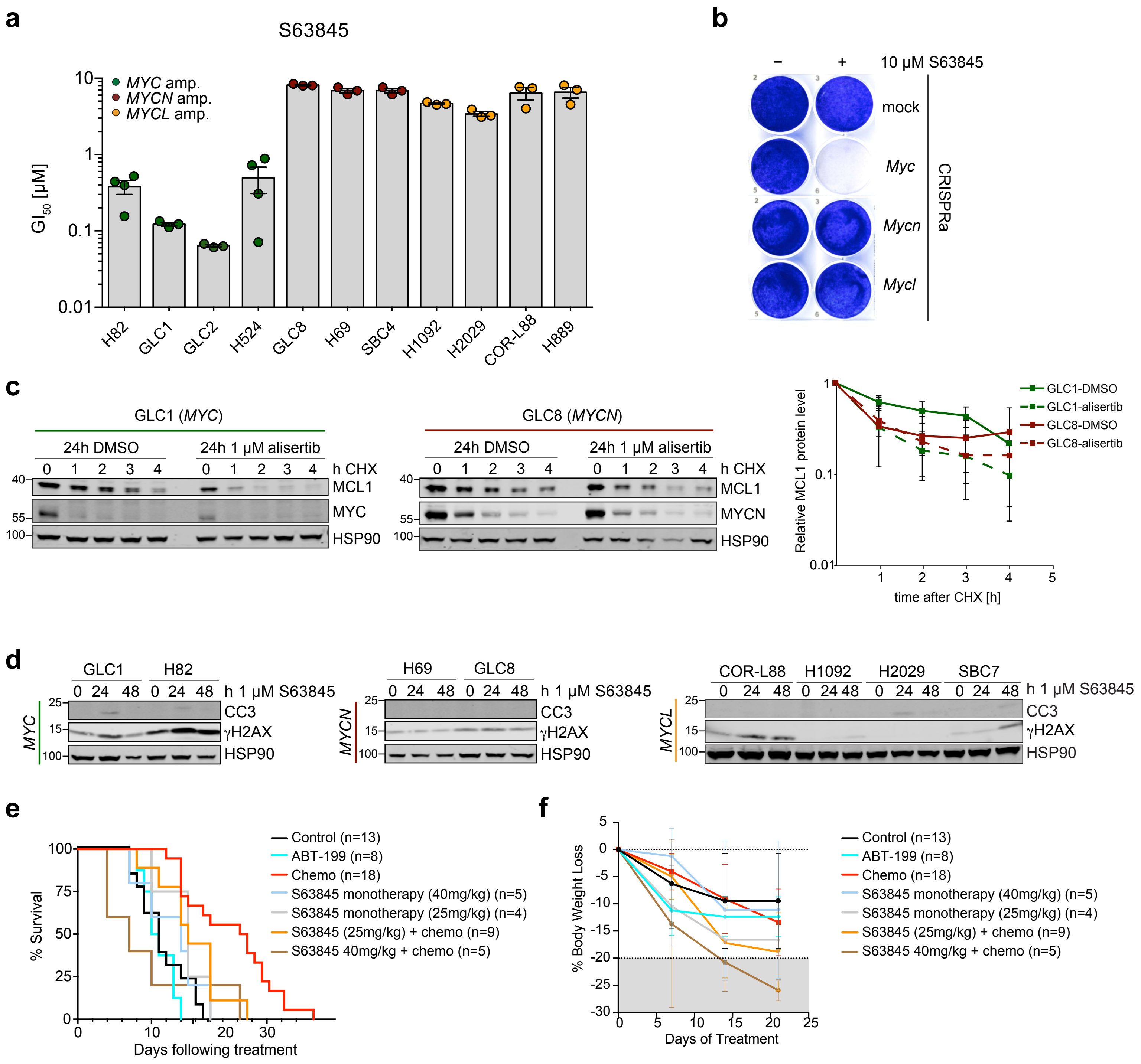

Supplementary Figure 3: MYC drives apoptotic priming and MCL1 dependency

(**a**): GI_{50} values of individual *MYC* paralog-amplified human SCLC cell lines (*MYC*-amplified: H82, GLC1, GLC2, H524; *MYCN*-amplified: GLC8, H69, SBC4; *MYCL*-amplified: H1092, H2029, COR-L88, H889) treated with MCL1 inhibitor (S63845) (n = 3; H82/H524 n = 4). Error bars indicate mean ± SEM.

(**b**): Crystal violet assay of *Myc* paralog-activated CRISPRa cells after treatment with 10 µM for 96 h.

(**c**): Western blot showing protein turnover of MCL1. *MYC*-amplified GLC1 and *MYCN*-amplified GLC8 cells were pre-treated 24 h with DMSO (control) or 1 µM alisertib followed by cycloheximide (CHX) treatment to block protein translation. MCL1 and MYC/MYCN protein levels were monitored hourly after CHX treatment. HSP90 was used as loading control (right). MCL1 protein amounts were quantified relative to HSP90 over-time during CHX treatment (n = 3).

(**d**): Western blot showing protein levels of cleaved caspase 3 (CC3) and γH2AX in *MYC* paralog-amplified human SCLC cell lines (n = 8, *MYC*-amplified: GLC1, H82; *MYCN*-amplified: H69, GLC8; *MYCL*-amplified: COR-L88, H1092, H2029, SBC7) treated with MCL1i (S63845). HSP90 was used as loading control.

(**e**): Kaplan-Meier survival analysis of RPM mice bearing MYC-driven SCLC after treatment with vehicle control (PBS, n = 13), chemotherapy (cisplatin/etoposide, n = 18), ABT-199 (n = 8), S63845 (n = 9), S63845 (40 mg/kg) + chemotherapy (n = 5), and S63845 (25 mg/kg) + chemotherapy (n = 9).

(**f**): Percent body weight change of RPM mice during treatment with the indicated treatment regimens.

Source data are provided as a Source Data file.

Supplementary Figure 4

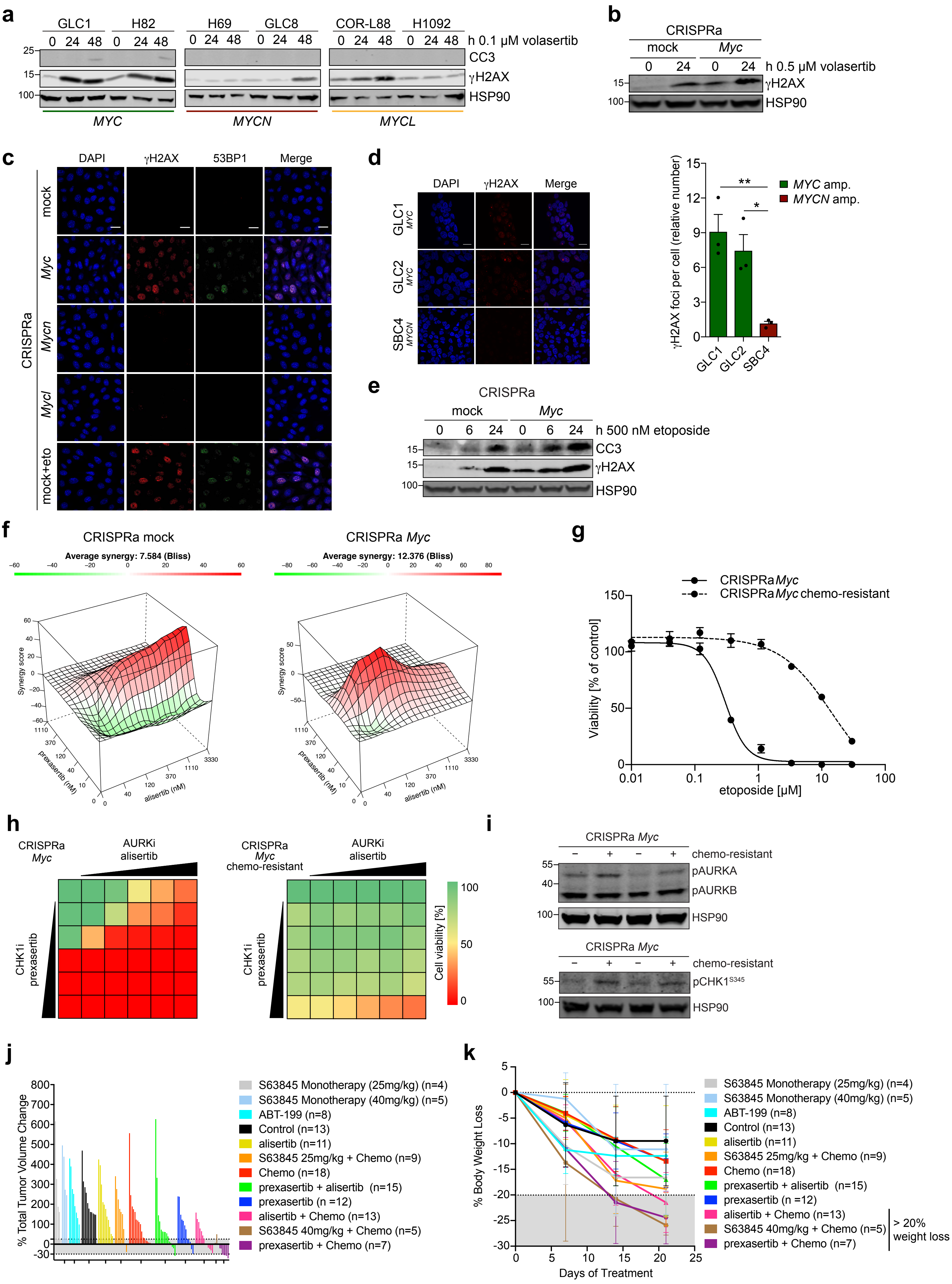

Supplementary Figure 4: MYC triggers a druggable DNA-damage response *in vivo*

(**a**): Western blot of *MYC* paralog-amplified human SCLC cell lines (n = 6) treated with 0.1 µM volasertib for the indicated time points. Cleaved caspase 3 (CC3) and γH2AX protein levels were monitored. HSP 90 was used as loading control.

(**b**): Western blot of *Myc*-activated and control CRISPRa cells treated with 0.5 µM volasertib for the indicated time points. γH2AX protein levels were monitored. HSP 90 was used as loading control.

(**c**): IF images of Fig. 5a including etoposide treatment of *Myc* paralog-activated CRISPRa cells as positive control for DNA damage induction showing DAPI (DNA), γH2AX (DDR activation) and 53BP1 (DNA double strand break) staining. Scale bar: 20 µm.

(**d**): (left) IF images showing γH2AX and DAPI staining in human SCLC cell lines GLC1, GLC2 (*MYC*-amplified) and SBC4 (*MYCN*-amplified). Scale bar: 20 µm. (right) Quantification of IF staining displaying number of γH2AX foci per cell. n = 30 cells. Error bars indicate mean ± SEM. Two-tailed unpaired t tests, **p < 0.01, *p < 0.05.

(**e**): Western blot showing cleaved caspase 3 (CC3) and γH2AX levels of mock control and *Myc*-activated CRISPRa cells treated with 500 nM etoposide for 0, 6 and 24 h. HSP90 was used as loading control.

(**f**): Display of Bliss synergy score of alisertib and prexasertib combination treatment of *Myc*-activated and mock control CRISPRa cells.

(**g**): Cell viability screening of etoposide-treated parental and chemo-resistant *Myc*-activated CRISPRa cells after 96 h (n = 3).

(**h**): Cell viability of parental and chemo-resistant *Myc*-activated CRISPRa cells subjected to combinatorial alisertib and prexasertib treatment for 96 h (concentration range 40 nM – 3.3 µM). Percentage of cell viability is displayed as indicated by the color scheme (n = 3).

(**i**): Western blot showing protein levels of pAURKA/B and pCHK1 in parental and chemo-resistant *Myc*-activated CRISPRa cells. HSP90 was used as loading control.

(**j**): Percentage of total tumor volume change of RPM mice at day 14 after treatment start with the indicated treatment regimens.

(**k**): Percent body weight change of RPM mice during treatment with the indicated treatment regimens.

Source data are provided as a Source Data file.

Supplementary Tables

Supplementary Table 1: IHC analysis of MYC and BCL2 protein expression of 49 SCLC patient samples

		**MYC IHC score**		
		**high**	**low**	**total**
**BCL2 IHC Score**	**high**	0 (0 %)	29 (59 %)	29 (59 %)
	**low**	2 (4 %)	18 (37 %)	20 (41 %)
	**total**	2 (4%)	47 (96 %)	49 (100 %)

Supplementary Table 2: Oligonucleotide sequences used in this study

Oligonucleotides (Primers)	
18S rRNA for	CGCCGCTAGAGGTGAAATTCT
18S rRNA rev	CGAACCTCCGACTTTCGTTCT
mouse Myc-for	CCTAGTGCTGCATGAGGAGA
mouse Myc-rev	TCTTCCTCATCTTCTTGCTCTTC
mouse Mycl-for	ACGGCACTCCTAGTCTGGAA
mouse Mycl-rev	CCACGTCAATCTCTTCACCTT
mouse Mycn-for	CCTTGAGCGACTCAGATGATGA
mouse Mycn-rev	GGACGCACAGTGATCGTGA
mouse Bcl2-for	GAACTGGGGGAGGATTGTGG
mouse Bcl2-rev	GCATGCTGGGGCCATATAGT
BCL2-promoter-bisulfite-for	GTTATGAAAATAAGGGTTGGAAAAG
BCL2-promoter-bisulfite-rev	ACAAAATCACCTATCTTCACAACAA
BCL2-promoter-ChIP-for	GGTGCCGAGCGCTAGAA
BCL2-promoter-ChIP-rev	GAACTTCGTAGCAGTCATCCT
ACTB-promoter-ChIP-for	GCCATAAAAGGCAACTTTCGG
ACTB-promoter-ChIP-rev	TCTTCCTCAATCTCGCTCTCG
M13-for	GTAAAACGACGGCCAG
M13-rev	CAGGAAACAGCTATGAC
DNTM3A-for	CAGCTTCCACGTTGCCTTCT
DNMT3a-rev	CATCTGCAAGCTGTCTCCCTTT
BCL2-for	CTGCACCTGACGCCCTTCACC
BCL2-rev	CACATGACCCCACCGAACTCAAAGA
**sgRNA Sequences**	
sgMyc#1	GAGGGCGGGGAAGCGAGAGG
sgMyc#2	AGGGAGAAAGAGAGATTCTC
sgMyc#3	GCTTTGGGAACTCGGGAGGG
sgMycl#1	CAGCGGAGAGAGGGGCGGGA
sgMycl#2	GTGGGGCCGGCCCCACCTCC
sgMycl#3	GGGGCTTGGGGACCAGGAGG
sgMycn#1	GGGAGAAAGGCAGAGGCAAG

sgMycn#2	CTGTGGCGCAGACGAGGGGG
sgMycn#3	GGCTGCGCTTTGAAGCTCGG
**siRNA sequences of SMARTpool**	
siMCL1#1	GGUUUGGCAUAUCUAAUAA
siMCL1#2	GAAGGUGGCAUCAGGAAUG
siMCL1#3	GAUUAUCUCUCGGUACCUU
siMCL1#4	CGAAGGAAGUAUCGAAUUU
siDNMT3A#1	GCAUUCAGGUGGACCGCUA
siDNMT3A#2	GCACUGAAAUGGAAAGGGU
siDNMT3A#3	CUCAGGCGCCUCAGAGCUA
siDNMT3A#4	GGGACUUGGAGAAGCGGAGS
