# Supplementary material for: MYC paralog-dependent apoptotic priming orchestrates a spectrum of vulnerabilities in small cell lung cancer
Source: Nat Commun. 2019 Aug 2;10:3485. doi: 10.1038/s41467-019-11371-x (PMC6677768; doi:10.1038/s41467-019-11371-x)
Supplement: Supplementary file 2 — Reporting Summary [file 41467_2019_11371_MOESM2_ESM.pdf]

nature research

Corresponding author(s): Dr. Martin L. Sos

Last updated by author(s): Jun 21, 2019

Reporting Summary

Nature Research wishes to improve the reproducibility of the work that we publish. This form provides structure for consistency and transparency in reporting. For further information on Nature Research policies, see Authors & Referees and the Editorial Policy Checklist.

Statistics

For all statistical analyses, confirm that the following items are present in the figure legend, table legend, main text, or Methods section.

n/a	Confirmed	
☐	☒	The exact sample size (*n*) for each experimental group/condition, given as a discrete number and unit of measurement
☐	☒	A statement on whether measurements were taken from distinct samples or whether the same sample was measured repeatedly
☐	☒	The statistical test(s) used AND whether they are one- or two-sided<br>*Only common tests should be described solely by name; describe more complex techniques in the Methods section.*
☒	☐	A description of all covariates tested
☐	☒	A description of any assumptions or corrections, such as tests of normality and adjustment for multiple comparisons
☐	☒	A full description of the statistical parameters including central tendency (e.g. means) or other basic estimates (e.g. regression coefficient) AND variation (e.g. standard deviation) or associated estimates of uncertainty (e.g. confidence intervals)
☐	☒	For null hypothesis testing, the test statistic (e.g. F, t, r) with confidence intervals, effect sizes, degrees of freedom and P value noted<br>*Give P values as exact values whenever suitable.*
☒	☐	For Bayesian analysis, information on the choice of priors and Markov chain Monte Carlo settings
☒	☐	For hierarchical and complex designs, identification of the appropriate level for tests and full reporting of outcomes
☐	☒	Estimates of effect sizes (e.g. Cohen's *d*, Pearson's *r*), indicating how they were calculated

Our web collection on statistics for biologists contains articles on many of the points above.

Software and code

Policy information about availability of computer code

Data collection	LICOR Image Studio (Version 5.2.5) - Immunoblot acquisition<br>BWA-MEM - Alignment of sequencing reads (WES)<br>STAR - Alignment of sequencing reads (RNAseq)<br>PerkinElmer Quantum FX - µCT imaging
Data analysis	LICOR Image Studio (Version 5.2.5) - Immunoblot analysis<br>Graphpad PRISM (Version 7.0e) - Analysis of viability screening, RNA expression (qRT-PCR), ChIP qPCR, GI50 calculation, statistical testing, proliferation kinetics, kaplan-meier survival, weight-loss monitoring, IF quantification<br>RStudio - data analysis, statistics, platform for NGS plugins (listed below)<br>SCLUST - Copy number analysis (WES)<br>RSEM - Gene expression quantification<br>DESeq2 - Differential gene expression calculation<br>Synergyfinder (R-package) - Calculation of Bliss synergy score<br>ImageJ - IF, IHC picture analysis<br>QUMA quantification tool for methylation - Quantification of CpG methylation<br>Analyze 11.0 (AnalyzeDirect) - µCT image processing

For manuscripts utilizing custom algorithms or software that are central to the research but not yet described in published literature, software must be made available to editors/reviewers. We strongly encourage code deposition in a community repository (e.g. GitHub). See the Nature Research guidelines for submitting code & software for further information.

Data

Policy information about availability of data

All manuscripts must include a data availability statement. This statement should provide the following information, where applicable:

- Accession codes, unique identifiers, or web links for publicly available datasets
- A list of figures that have associated raw data
- A description of any restrictions on data availability

All data supporting the findings in this study are available from the corresponding author upon reasonable request. The primary data underlying the graphs are provided in the Source Data File. RNAseq data generated in this study have been deposited in EBI Array Express (https://www.ebi.ac.uk/arrayexpress/) with the accession codes E-MTAB-7411 and E-MTAB-7412.

Field-specific reporting

Please select the one below that is the best fit for your research. If you are not sure, read the appropriate sections before making your selection.

☒ Life sciences ☐ Behavioural & social sciences ☐ Ecological, evolutionary & environmental sciences

For a reference copy of the document with all sections, see nature.com/documents/nr-reporting-summary-flat.pdf

Life sciences study design

All studies must disclose on these points even when the disclosure is negative.

Sample size	Sample sizes were not determined a priori. Generally accepted samples sizes were used, with reproducible differences between conditions indicating that the sample size is sufficient.
Data exclusions	No data were excluded from the analyses.
Replication	Experimental findings were reproduced with at least three biological replicates. All replicates successfully validated the experimental findings.
Randomization	For animal experiments, littermates were randomly allocated to treatment groups.
Blinding	For animal studies, blinding was not applicable since dosing regimens of mice were not compatible with blinding. IHC studies were performed by two independent pathologists in a blinded fashion.

Reporting for specific materials, systems and methods

We require information from authors about some types of materials, experimental systems and methods used in many studies. Here, indicate whether each material, system or method listed is relevant to your study. If you are not sure if a list item applies to your research, read the appropriate section before selecting a response.

Materials & experimental systems

n/a	Involved in the study	
☐	☒	Antibodies
☐	☒	Eukaryotic cell lines
☒	☐	Palaeontology
☐	☒	Animals and other organisms
☒	☐	Human research participants
☒	☐	Clinical data

Methods

n/a	Involved in the study	
☒	☐	ChIP-seq
☒	☐	Flow cytometry
☒	☐	MRI-based neuroimaging

Antibodies

Antibodies used

Immunoblot & IF
MYC (#9402, Cell Signaling Technology)
MYCN (sc-53993, clone B8.4.B, Santa Cruz Biotechnology)
BCL2 (#2872, Cell Signaling Technology)
BIM (#2933, clone C34C5, Cell Signaling Technology)
BAD (#610391, clone 48, BD Biosciences)
BCL-xL (#2764, clone 54H6, Cell Signaling Technology)
HA (#3724, clone C29F4, Cell Signaling Technology)
MIZ1 (clone 10E2, Elmar Wolf, Würzburg)
MIZ1 (sc-136985, clone B10, Santa Cruz Biotechnology)
ASCL1 (#556604, Clone 24B72D11.1, BD Biosciences)

MCL1 (sc-819,, clone S19, Santa Cruz Biotechnology)
MCL1 (#94296, clone D2W9E, Cell Signaling Technology)
γH2AX (#05-636, clone JBW301, Merck)
53BP1 (MAB3802, clone BP13, Merck)
Cleaved Caspase 3 – CC3 (#9664, clone 5A1E, Cell Signaling Technology)
HSP90 (ADI-SPA-835, clone 16F1, Enzo Life Sciences)
HSP90 (#4877, clone C45G5, Cell Signaling Technology)
ACTIN (#A2066, Sigma)
Alexa Fluor-488 (A11029, Thermo Fisher Scientific)
Alexa Fluor-647 (A32733, Thermo Fisher Scientific)
pAURKA/B/C (#2914, Cell Signaling Technology)
pCHK1S345 (#2341, Cell Signaling Technology)

IHC:
BCL2 (M0887, clone 124, Dako)
MYC (ab32072, clone Y69, Abcam)

ChIP:
MYC (ab56, clone 9E11, Abcam)
MYCN (sc-53993, clone B8.4.B, Santa Cruz Biotechnology)
DNMT3a (ab2850, Abcam)
MIZ1 (clone 10E2, provided by Elmar Wolf, Würzburg)
mouse IgG (sc-2025, Santa Cruz Biotechnology)

Validation

All antibodies are well-validated by the manufacturer and are widely used in the scientific community. For all antibodies used, we detected a strong signal and specific bands at the expected size in mouse and human cell lines.

Eukaryotic cell lines

Policy information about cell lines

Cell line source(s)

Cell lines were purchased from ATCC with following exceptions: GLC8, GLC1, GLC2, SBC4, and SBC7 were gifts from Prof. Thomas; COR-L303 and COR-L88 were purchased from European Collection of Authenticated Cell Cultures (ECACC, Public Health England). Murine SCLC cell lines RP and MEFs were gifts from Prof. Reinhardt.

Authentication

Cell lines were authenticated by STR profiling at the University of Utah DNA sequencing core facility or at the Institute for Forensic Medicine of the University Hospital of Cologne.

Mycoplasma contamination

All cell lines were routinely tested for mycoplasma contamination and were negative.

Commonly misidentified lines
(See ICLAC register)

None of the cell lines used in this study is present in the database of commonly misidentified cell lines.

Animals and other organisms

Policy information about studies involving animals; ARRIVE guidelines recommended for reporting animal research

Laboratory animals

Animal experiments were performed with male and female Igs2(tm1[CAG-Myc*T58A/luc]Wrey, Trp53(tm1Brn), Rb1(tm3Tyj)/OlvrJ also known as Rb1fl, Trp53fl, MycLSL (RPM) mice. At 6–8 weeks of age, anesthetized mice were infected with Adene-Cre virus by intratracheal instillation to trigger condidtional Rb1, Trp53 knockout and MycLSL expression.

Wild animals

Study did not involve wild animals.

Field-collected samples

Study did not involve field-collected samples.

Ethics oversight

Animal experiments were approved by the Institutional Animal Care and Use Committee (IACUC) of the Huntsman Cancer Institute (HCI).

Note that full information on the approval of the study protocol must also be provided in the manuscript.
